# Synergistic power of functional foods and exercise in colorectal cancer control: targeting metabolism, mitochondrial function, redox homeostasis, exercise performance, neuroimmune signaling, and brain–gut axis crosstalk

**DOI:** 10.3389/fnut.2025.1640092

**Published:** 2025-09-03

**Authors:** Mengliang Cheng, Yunlong Li, Danqing Liang, Chuanzhong Wu

**Affiliations:** ^1^Guangdong Justice Police Vocational College, Guangdong, China; ^2^Xianda College of Economics and Humanities Shanghai International Studies University, Shanghai, China

**Keywords:** functional foods, exercise, gastrointestinal cancer, metabolism, mitochondrial function

## Abstract

Gastrointestinal (GI) cancers—including colorectal, gastric, esophageal, and pancreatic malignancies—are among the most prevalent and lethal cancers globally. Despite advancements in treatment, the prognosis for many patients remains poor, highlighting the urgent need for integrative and preventive approaches. Functional foods, rich in bioactive compounds such as polyphenols, flavonoids, carotenoids, and omega-3 fatty acids, possess potent antioxidant, anti-inflammatory, and antitumor properties. Meanwhile, exercise has emerged as a non-pharmacological intervention capable of modulating systemic inflammation, enhancing immune responses, and improving quality of life in cancer patients. This review critically examines the synergistic impact of consuming functional foods and engaging in regular physical activity on the molecular pathways underlying the initiation and progression of GI cancer. Emphasis is placed on key signaling cascades, which are implicated in oxidative stress, inflammation, cell proliferation, apoptosis, and metastasis. Furthermore, the role of neurotrophic factors—particularly brain-derived neurotrophic factor—is explored, revealing their potential as molecular links between gut–brain axis modulation, cancer pathophysiology, and exercise-induced neuroprotection. By integrating current preclinical and clinical evidence, this review highlights the potential of combining functional nutrition with exercise as a holistic, personalized strategy for preventing and managing GI cancers. Such approaches may not only target tumor biology but also improve cognitive function, mood, and overall patient wellbeing, paving the way for precision lifestyle medicine in oncology.

## 1 Introduction

Colorectal cancer (CRC) remains one of the most prevalent and deadly malignancies worldwide, ranking as the fourth leading cause of cancer-related mortality globally and the second most common cancer in Europe (1). Despite advances in screening, surgical techniques, and chemotherapeutic regimens, CRC continues to pose a significant public health burden due to its high recurrence rates, treatment-related complications, and associated comorbidities such as cancer cachexia, depression, and functional decline. Moreover, conventional therapies often fail to address the broader physiological and psychosocial impacts of the disease, prompting a need for integrative, supportive strategies (1, 2). In recent years, increasing attention has been directed toward the role of functional foods and physical exercise as complementary approaches to conventional CRC treatment. Functional foods, defined as foods or food components that provide health benefits beyond basic nutrition, are rich in bioactive compounds such as curcumin (CUR), resveratrol, epigallocatechin gallate (EGCG), quercetin, and genistein (GEN) (3). These polyphenols and flavonoids have demonstrated anticancer properties through various mechanisms, including anti-inflammatory, antioxidant, pro-apoptotic, and epigenetic modulation. Their ability to target key signaling pathways involved in CRC progression, such as the Wnt/β-catenin, PI3K/Akt, nuclear factor kappa B (NF-κB) pathways, and transforming growth factor-beta (TGF-β), positions them as promising agents for chemoprevention and adjunctive therapy (3, 4).

Simultaneously, physical exercise, particularly aerobic and resistance training, has emerged as an effective non-pharmacological intervention that can improve CRC patient outcomes (5). Exercise not only enhances functional capacity and quality of life but also mitigates treatment-induced complications, reduces systemic inflammation, and preserves lean body mass during and after therapy. The concept of prehabilitation, which integrates structured physical training before surgical intervention, has shown promise in optimizing recovery and minimizing postoperative decline. Mechanistically, physical activity enhances immune surveillance by increasing cytotoxic T cell activity and natural killer cell function, modulates insulin signaling by improving insulin sensitivity and reducing circulating insulin-like growth factors, and positively alters gut microbiota composition, promoting anti-inflammatory microbial profiles. These pathways contribute to the suppression of tumorigenesis and the improvement of systemic homeostasis in CRC patients (5).

Emerging research has also highlighted the relevance of the gut–brain axis and neuroimmune signaling in the development and progression of CRC. Disruptions in gut microbiota composition, intestinal permeability, and neurotrophic signaling may influence systemic inflammation, immune responses, and even cognitive function in cancer patients. Both functional foods and physical activity have demonstrated potential to modulate these pathways, suggesting a broader, systems-level impact that extends beyond tumor biology alone. This study aims to explore the synergistic potential of functional foods and exercise in the context of CRC, focusing on their combined ability to influence molecular, physiological, and psychological outcomes. By integrating insights from experimental and clinical studies, we highlight how lifestyle-based interventions may serve as powerful tools in enhancing CRC prevention, treatment, and survivorship. This review synthesizes current mechanistic and clinical evidence to propose an integrative framework for CRC management.

## 2 Search methodology

This narrative review is based on a comprehensive literature search conducted in PubMed, Scopus, Web of Science, and Google Scholar up to March 2025. We used combinations of keywords including “colorectal cancer,” “functional foods,” “polyphenols,” “curcumin,” “resveratrol,” “EGCG,” “genistein,” “exercise,” “epigenetics,” “microRNA (miRNA),” and “chemoresistance.” Studies were included if they reported original experimental (*in vitro* or *in vivo*), preclinical, or clinical data relevant to the effects of functional foods or exercise on colorectal cancer progression, treatment, or prevention. We prioritized peer-reviewed English-language publications, and excluded editorials, conference abstracts, or studies lacking mechanistic insight. Preference was given to studies published within the last 10 years, although earlier seminal studies were also considered.

## 3 Polyphenols in CRC: epigenetic and molecular targets

### 3.1 Curcumin affects cancer cells through epigenetic regulation

#### 3.1.1 Chemical properties and bioavailability

Turmeric, obtained from *Curcuma longa* L., a member of the ginger family, typically contains 2–9% curcuminoids by dry weight. The primary bioactive compound, curcumin, is a polyphenol with the chemical name 1,7-bis(3,4-dimethoxyphenyl)-1,6-heptadiene-3,5-dione (also known as diferuloylmethane) and with the molecular formula C_2_1H_2_0O6. Commercial curcumin formulations primarily consist of three curcuminoids: curcumin I (77%), demethoxycurcumin (DMC, 17%), and bisdemethoxycurcumin (BDMC, 3%) (6–9). Curcumin is chemically unstable, particularly in aqueous environments at physiological pH, where it undergoes rapid degradation (half-life of 4–8 min), yielding various breakdown products. These degradation products include (a) alkaline hydrolysis products such as ferulic acid, vanillin, and ferulic methane, resulting from cleavage of the central carbon chain and (b) oxidative cyclization products, primarily bicyclopentadione derivatives, generated via autoxidation (10–12). Notably, autoxidation is now recognized as the dominant degradation pathway, while alkaline hydrolysis plays a minor role. Curcumin is classified as “Generally Recognized As Safe” by the U.S. Food and Drug Administration, indicating a high safety margin even at elevated doses, as supported by established no-observed-adverse-effect levels (13, 14). It exhibits a wide range of pharmacological properties, including antioxidant, antibacterial, anticancer, antitumor, lipid-lowering, and neuroprotective activities. Due to its multifaceted bioactivity, curcumin is widely utilized in various sectors, including pharmaceuticals, nutraceuticals, cosmetics, and the development of functional foods (15). A key way in which antitumor effects operate is through the suppression of tumor invasion and migration, the promotion of apoptosis in tumor cells, and the inhibition of several cell signaling pathways. Research has shown that the antitumor activity of curcumin is linked to epigenetic mechanisms (also summarized in Table 1). This suggests that curcumin could serve as a potential epigenetic modulator that affects the progression of tumor-related diseases.

**Table 1:** A summary of studies investigating the effects of curcumin on different epigenetic processes.

**Epigenetic process**	**Model**	**Result(s)**	**References**
Histone modification	*In vitro*	Curcumin reduces lysine methylation and downregulates EZH2, G9a, and MLL1 methyltransferases	(20)
DNA methylation	*In vivo*	176 regions that were hypomethylated in cancer were remethylated after curcumin treatment, including the *Tnf* gene	(16)
		*In vitro*	Curcumin treatment led to the re-expression of DLEC1 in HT29 colon cancer cells	(17)
	*In vitro*	Epigenetically reactivating CDX2 through demethylation of its promoter and inhibiting EMT	(19)
microRNAs	*In vitro*	Upregulation of tumor-suppressive miR-34a and downregulation of miR-27a in colorectal cancer cells	(23)
	*In vivo*	CUR suppressed tumor growth, which corresponded with alterations in the expression of miR-34a and miR-27a	(23)
	*In vitro*		(33)
	*In vivo*		(33)
	*In vitro*	CUR decreased the expression of miR-27a and increased the expression of ZBTB10	(28)
	*In vitro*	Curcumin upregulated miR-134-5p and downregulated CDCA3	(29)
	*In vivo*	Curcumin treatment significantly reduced tumor growth in mice, accompanied by increased miR-134-5p expression and reduced CDCA3 and CDK1 levels	(29)
	*In vitro*	Curcumin reverses cisplatin resistance by upregulating miR-137, which in turn downregulates GLS and impairs glutamine metabolism	(32)
	*In vitro*	Curcumin inhibits EMT by upregulating miR-200c, which in turn suppresses EPM5	(25)
	*In vitro*	Curcumin downregulates circHN1, it relieves suppression on miR-302a-3p, which in turn targets and downregulates PIK3R3	(26)
	*In vitro*	Curcumin sensitizes resistant CRC cells to oxaliplatin through modulation of miR-409-3p, which in turn downregulates ERCC1	(34)

#### 3.1.2 DNA methylation effects

**Figure 1:**
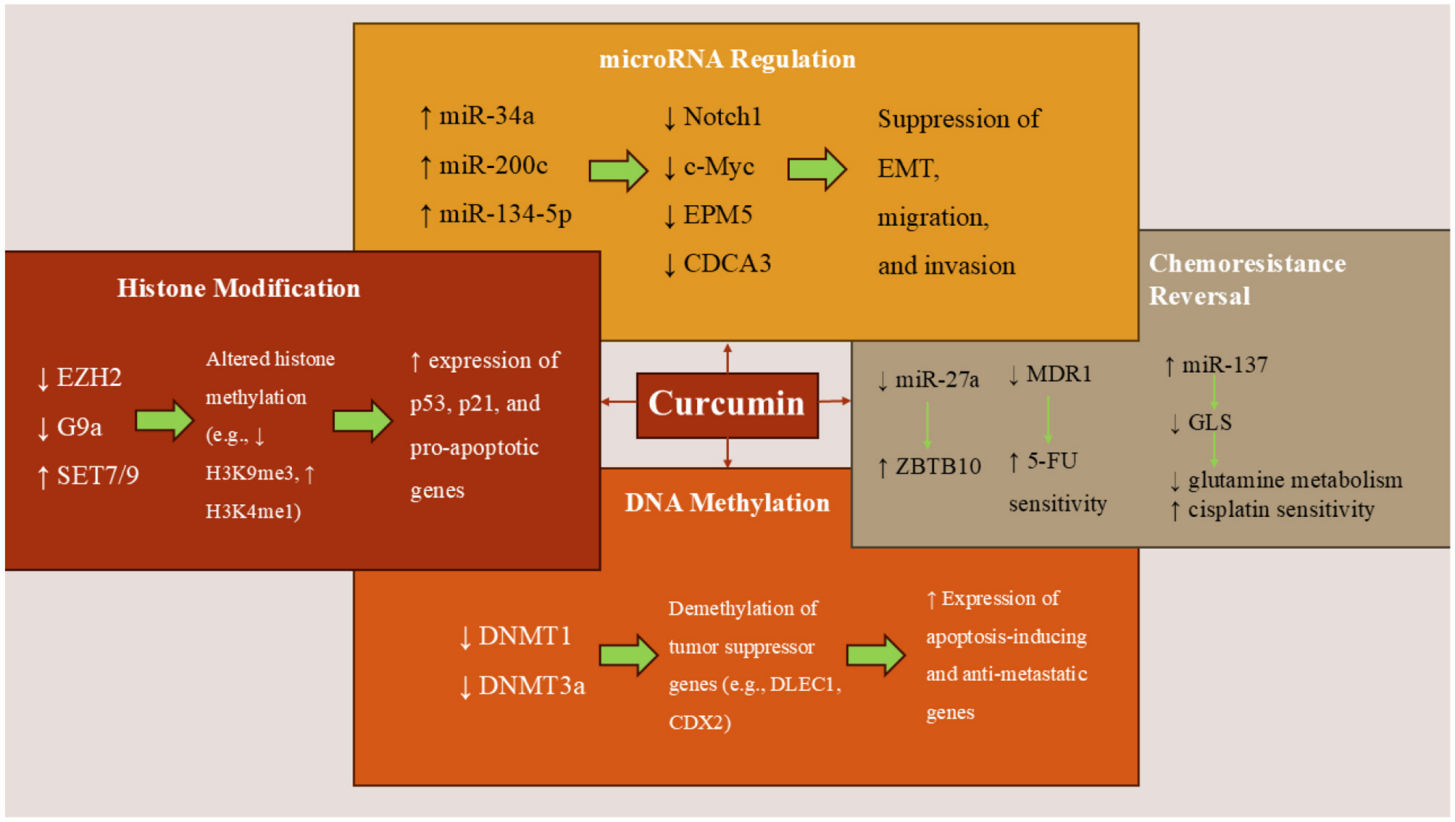
Curcumin's epigenetic mechanisms in colorectal cancer. Curcumin modulates key epigenetic regulators in CRC cells by inhibiting DNMTs and HDACs, thereby reactivating tumor suppressor genes. It alters histone modifications, upregulates tumor-suppressive miRNAs (e.g., miR-34a and miR-200c), and downregulates oncogenic miRNAs (e.g., miR-27a), thereby inhibiting EMT, proliferation, and metastasis. Curcumin also enhances chemosensitivity by targeting miRNA-mediated pathways linked to drug resistance and glutamine metabolism.

Curcumin (CUR) is approved to have influences on several epigenetic processes in CRC cells (Figure 1). Modulating DNA methylation is one of the most studied processes in this field. CUR is shown to significantly alter the epigenetic landscape in a mouse model of colitis-associated CRC. Specifically, curcumin reversed abnormal DNA methylation patterns, demethylating tumor suppressor genes and methylating oncogenes. These epigenetic changes were associated with corresponding shifts in gene expression, especially in pathways related to inflammation, cell proliferation, and apoptosis. The results suggest curcumin's cancer-preventive effects may be driven by restoring normal DNA methylation and transcriptional regulation. Curcumin altered DNA methylation patterns in mice with azoxymethane (AOM) + dextran sulfate sodium (DSS)-induced colon cancer by increasing methylation at some genomic regions and decreasing it at others. Notably, 176 regions that were hypomethylated in cancer were remethylated after curcumin treatment, including the tumor necrosis factor (TNF) gene, suggesting a reversal of tumor-associated epigenetic changes. A broader analysis revealed over 2,700 regions with reduced methylation in cancer and over 1,500 regions that curcumin remethylated, highlighting its ability to restore a more normal epigenetic state (16).

To assess the impact of curcumin on DNA methylation, another study used three CRC cell lines—a harbor cancer therapy group cell line 116 (HCT116), a human tumor cell line 29 (HT29), and RKO—which were exposed to curcumin treatment (17). Cells treated with 5-aza2′-deoxycytidine (5-aza-CdR) served as a positive control for changes in DNA methylation, while trichostatin A (TSA)-treated cells acted as a negative control. The evaluation of global methylation and alterations in gene expression was conducted through the analysis of long interspersed nuclear elements-1 (LINE-1) repeat elements, DNA promoter methylation microarrays, and gene expression arrays. Validation of the findings was carried out using independent microarrays, quantitative bisulfite pyrosequencing, and quantitative polymerase chain reaction (qPCR). As anticipated, genome-wide methylation microarrays indicated a significant reduction in DNA methylation in cells treated with 5-aza2′-deoxycytidine (5-aza-CdR), with a mean β-value of 0.12 (17). In contrast, curcumin-treated cells exhibited non-significant changes in mean β-values. When compared to mock-treated cells, the alterations in DNA methylation induced by curcumin were observed to be time-dependent. Unlike the broad and non-specific global hypomethylation observed with 5-aza-CdR, curcumin treatment led to changes in methylation at specific, partially methylated loci rather than at fully methylated guanine (CpG) sites. These alterations in DNA methylation were corroborated by corresponding changes in gene expression, affecting both upregulated and downregulated genes across various CRC cell lines (17). Another study focused on the methylation status of the deleted in lung and esophageal cancer 1 (DLEC1) gene, which is often silenced in CRC due to promoter hypermethylation (17). They found that curcumin treatment led to the re-expression of DLEC1 in HT29 colon cancer cells. The reactivation of DLEC1 was associated with decreased DNA methylation at its promoter region and alterations in histone modifications, including increased acetylation of histone H3 and H4, which are markers of active transcription. Restoration of DLEC1 expression resulted in reduced anchorage-independent growth of HT29 cells, indicating suppressed tumorigenic potential (17).

Epithelial–mesenchymal transition (EMT) is a key process in CRC progression and metastasis, characterized by the loss of epithelial markers (e.g., E-cadherin) and the gain of mesenchymal markers (e.g., vimentin and ZEB1), enhancing cancer cell migration and invasion (18). EMT is regulated by multiple signaling pathways, including TGF-β, Wnt/β-catenin, and inflammatory cytokines, as well as by various microRNAs [e.g., microRNA-200c (miR-200c) and miR-34a]. Because of its central role in metastasis, many natural compounds, including resveratrol and curcumin, have been studied for their ability to reverse EMT or induce mesenchymal-to-epithelial transition (MET), thereby suppressing tumor invasiveness (18). Another study investigated the effects of curcumin on EMT and DNA methylation in SW480 CRC cells (19). Treatment with increasing concentrations of curcumin (0.1–0.4 μmol/L) did not significantly affect cell viability, allowing for further molecular analysis at these concentrations. Curcumin was found to reduce the expression of DNA methyltransferase 1 (DNMT1) and DNMT3a in a dose-dependent manner, which correlated with a significant decrease in methylation at the promoter region of the cdx2 gene. This epigenetic change led to enhanced expression of CDX2 and downstream suppression of the Wnt3a/β-catenin signaling pathway, as evidenced by reduced nuclear translocation of β-catenin. Furthermore, curcumin treatment inhibited EMT markers, including N-cadherin, vimentin, Snail1, Twist, and Wnt3a, while upregulating E-cadherin expression in a concentration-dependent fashion. Immunofluorescence confirmed reduced nuclear β-catenin levels, consistent with the repression of Wnt signaling (19). These findings suggest that curcumin inhibits EMT in CRC by epigenetically reactivating CDX2 through demethylation of its promoter, thereby downregulating the CDX2/Wnt3a/β-catenin axis and restoring epithelial characteristics. This mechanism highlights the potential of curcumin as a therapeutic agent targeting both epigenetic regulation and metastasis-related signaling pathways in CRC (19).

#### 3.1.3 Histone modification effects

Recent research also shows that histone modifications can be regulated by CUR (Figure 1). A study on colon cancer cell lines that carry wild-type p53 (wtp53) or mutant p53 (mutp53) shows that curcumin alters the methylation patterns and expression levels of various methyltransferases in colon cancer cells with both wild-type p53 (wtp53) and mutant p53 (mutp53) (20). It impacted methyltransferases associated with repressive markers, such as enhancer-of-zeste homolog 2 (EZH2) and a histone methyltransferase (G9a), as well as those linked to activation markers, such as mixed-lineage leukemia 1 (MLL1) protein and Su(var)3-9, enhancer-of-zeste, and trithorax (SET) domain-containing protein 7/9 (SET7/9). Notably, G9a was downregulated at the transcriptional level in wtp53 HCT116 cells, whereas in mutp53 SW480 cells, the downregulation occurred post-transcriptionally. Additionally, curcumin demonstrated increased cytotoxicity, which was associated with its more pronounced demethylating effects in HCT116 cells. These cells exhibited higher baseline levels of lysine methylation and showed greater sensitivity to the inhibition of EZH2, MLL1, and G9a (20). A study investigates the role of the histone demethylase Jumonji domain-containing protein 2C (JMJD2C) in CRC and explores the potential of curcuminoids as inhibitors of this enzyme (21). The research demonstrated that JMJD2C is overexpressed in HCT116 colon cancer cells and contributes to their proliferation. Silencing JMJD2C led to reduced cell growth, indicating its pro-growth role in CRC. The study identified that curcuminoids, compounds derived from turmeric, can inhibit the activity of JMJD2C (21). This inhibition was associated with a decrease in the proliferation of colon cancer cells, suggesting that curcuminoids may exert anticancer effects by targeting JMJD2C. By inhibiting JMJD2C, curcuminoids alter histone methylation patterns, leading to changes in gene expression that suppress tumor growth. This highlights the potential of curcuminoids as epigenetic modulators in cancer therapy. The findings suggest that JMJD2C plays a significant role in the growth of CRC cells and that curcuminoids can inhibit this enzyme, leading to suppressed tumor proliferation (21).

#### 3.1.4 miRNA regulation

Besides DNA methylation and histone modification, numerous research studies have confirmed that CUR can also alter the expression of miRNAs in CRC cells (Figure 1). A recent systematic review in 2024 detected that curcumin was found to upregulate several tumor-suppressive miRNAs, including miR-497, miR-200c, miR-200b, miR-409-3p, miR-34, miR-126, miR-145, miR-206, miR-491, miR-141, miR-429, miR-101, and miR-15a. It also led to the downregulation of oncogenic miRNAs such as miR-21, miR-155, miR-221, miR-222, miR-17-5p, miR-130a, miR-27, and miR-20a (22). Focusing on the impacts of CUR on miRNA expression shows that treatment with this agent led to the upregulation of tumor-suppressive miRNAs, specifically miR-34a and miR-27a (23, 24). These miRNAs are known to play critical roles in regulating genes involved in cell proliferation, apoptosis, and metastasis. The upregulation of miR-34a and miR-27a was associated with the downregulation of their target oncogenes, including Notch1 and c-Myc, which are implicated in CRC progression. These findings suggest that curcumin may exert chemopreventive effects in CRC by modulating the expression of specific miRNAs, leading to the suppression of oncogenic pathways. Findings are also validated by an *in vivo* study (23).

Metastatic CRC remains a major clinical challenge, with conventional treatments often accompanied by significant toxicity. This has driven the search for alternative therapeutic strategies, among which natural compounds, like curcumin, have drawn considerable interest due to their low toxicity and potent antitumor effects. To investigate the molecular mechanisms underlying curcumin's antimetastatic activity, one study employed qPCR and Western blotting to detect changes in gene and protein expression (25). They found that curcumin treatment significantly altered EMT-related gene expression, leading to reduced migration and invasion in CRC cells. Mechanistic investigations revealed that curcumin's suppression of EMT depended on the upregulation of miR-200c. The study identified epimorphin 5 (EPM5) as a direct target of miR-200c, and its high expression was enriched in consensus molecular subtype (CMS) 4, a poor-prognosis CRC subtype. Notably, overexpression of EPM5 alone induced EMT, while EPM5 knockdown reversed EMT, confirming its essential role in metastasis (25). Furthermore, downregulation of EPM5 was required for curcumin's ability to inhibit EMT, migration, and invasion. Clinically, higher levels of EPM5 correlated with advanced TNM stage and poorer overall survival. This study provides the first evidence that curcumin inhibits EMT in CRC by upregulating miR-200c, which in turn suppresses EPM5, a key promoter of EMT and metastasis. These findings suggest that curcumin may serve as a promising therapeutic agent for preventing or delaying CRC progression, particularly by targeting metastatic mechanisms in aggressive CRC subtypes (25). In another study, curcumin also significantly inhibited the growth and aggressiveness of CRC cells in a dose-dependent manner, including suppression of EMT-related processes (26). The circular RNA (circRNA) circular hematological and neurological expressed 1 (circHN1) gene was upregulated in CRC tissues, and its overexpression reversed the inhibitory effects of curcumin on cancer cell proliferation and migration. Conversely, silencing circHN1 enhanced curcumin's antitumor effect in both *in vitro* and *in vivo* models (26). Mechanistically, circHN1 functioned as a molecular sponge for miR-302a-3p, sequestering it and reducing its activity. MiR-302a-3p was found to directly target PIK3R3, a key component in the phosphoinositide 3-kinase (PI3K) signaling pathway. Inhibition of miR-302a-3p or overexpression of PIK3R3 diminished curcumin's efficacy, confirming the functional importance of the circHN1/miR-302a-3p/PIK3R3 axis in mediating its anti-CRC effects (26). This study reveals a novel mechanism by which curcumin inhibits CRC progression: by downregulating circHN1, it relieves suppression on miR-302a-3p, which in turn targets and downregulates PIK3R3, leading to reduced malignancy. These findings provide strong evidence that the circHN1/miR-302a-3p/PIK3R3 pathway plays a crucial role in CRC development and is an important target of curcumin action, supporting its further development as a therapeutic agent in CRC (26).

Another study also investigated the role of the p53/miR-34 axis in mediating the anticancer effects of curcumin in CRC (27). Using isogenic CRC cell lines genetically deficient for p53, miR-34a, and/or miR-34b/c, researchers showed that curcumin induced apoptosis and senescence, and inhibited migration and invasion, even in the absence of functional p53 (27). These effects were found to be driven by the activation of the Kelch-like ECH-associated protein 1 (KEAP1)/nuclear factor erythroid 2-related factor 2 (NRF2)/antioxidant response element (ARE) antioxidant pathway, through the generation of reactive oxygen species (ROS). Curcumin upregulated miR-34a and miR-34b/c expression in a ROS- and NRF2-dependent, but p53-independent, manner. Further analysis revealed that NRF2 directly binds to antioxidant response elements (AREs) in the promoter regions of miR-34a/b/c, leading to their transcriptional activation. Additionally, curcumin was able to counteract the repression of these miRNAs induced by interleukin-6 (IL-6) and hypoxia (27). Deletion of miR-34a and miR-34b/c markedly reduced curcumin's ability to induce apoptosis and senescence, and abolished its inhibitory effects on migration and invasion, confirming the central role of these miRNAs in curcumin's antitumor mechanisms. *In vivo*, intravenous injection of CRC cells into non-obese diabetic/severe combined immunodeficiency (NOD/SCID) mice showed that curcumin prevented lung metastasis formation in a miR-34a-dependent manner, suggesting curcumin induces mesenchymal-to-epithelial transition (MET) (27). Moreover, curcumin enhanced the efficacy of 5-fluorouracil (5-FU) even in cells lacking both p53 and miR-34a/b/c, indicating its potential to improve chemotherapy response in treatment-resistant CRC. Overall, the findings demonstrate that curcumin activates the KEAP1/NRF2/miR-34a/b/c axis, which mediates its tumor-suppressive effects, offering a novel strategy to activate miR-34 genes in CRC therapeutically (27).

#### 3.1.5 Reversal of chemoresistance

In another study, the effects of curcuminoids on CRC were investigated using two human colon cancer cell lines, human tumor 29 (HT29) and SW480, alongside normal CCD-18Co—a human colon fibroblast cell line derived from normal colon tissue (28). Curcuminoids, which had been previously analyzed by HPLC, were applied at concentrations ranging from 2.5 to 10 μg/mL. The researchers assessed changes in gene and protein expression using RT-PCR, transfection with expression constructs, and Western blotting. The results showed that curcuminoids preferentially inhibited the proliferation of the cancer cell lines HT29 and SW480, while having a milder effect on the growth of the normal CCD-18Co cells. Notably, curcuminoid treatment also enhanced the anticancer activity of 5-fluorouracil (5-FU), a commonly used chemotherapeutic agent. This enhancement was linked to the downregulation of the multidrug resistance 1 (MDR1) gene, which is associated with multidrug resistance (28). Mechanistically, the study found that curcuminoids suppressed the expression of transcription factors specificity protein 1 (Sp1), Sp3, and Sp4, as well as several Sp-regulated oncogenic genes in SW480 cells. This suppression was associated with a significant decrease in microRNA-27a (miR-27a) levels and a corresponding increase in zinc finger and BTB domain containing 10 (ZBTB10), a known target of miR-27a. ZBTB10 functions as a transcriptional repressor of Sp family proteins, suggesting that curcuminoids disrupt the miR-27a–ZBTB10–Sp1/3 axis (28). Further analysis revealed that the action of curcuminoids involved the generation of ROS, which played a critical role in modulating this regulatory pathway. Functional validation using RNA interference and ZBTB10 overexpression confirmed that MDR1 expression is regulated by Sp1 and Sp3, and that interference with the miR-27a–ZBTB10–Sp axis is a key mechanism through which curcuminoids exert their anticancer effects (28). Overall, the study provides evidence that curcuminoids suppress colon cancer cell growth and improve chemosensitivity by epigenetically targeting a ROS-dependent regulatory circuit involving miR-27a, ZBTB10, and Sp transcription factors, ultimately reducing the expression of MDR1 and other Sp-regulated genes (28).

In another study using HCT116 and SW620 colon cancer cell lines, researchers investigated the molecular mechanism underlying curcumin's anticancer activity, with a particular focus on the miR-134-5p/CDCA3/CDK1 signaling pathway (29). Quantitative RT-PCR was used to measure the expression levels of miR-134-5p and CDCA3, while Western blot analysis assessed the levels of key regulatory proteins, including c-Myc, MMP9, CDCA3, and CDK1. Besides, *in vivo*, SW620 cells were injected into mice to establish xenograft tumors, allowing for evaluation of curcumin's effects on tumor progression (29). The results demonstrated that curcumin inhibited cell proliferation and invasion while inducing apoptosis in both HCT116 and SW620 cells. Mechanistically, curcumin upregulated miR-134-5p and downregulated CDCA3. Functional studies revealed that inhibition of miR-134-5p or overexpression of CDCA3 reversed the suppressive effects of curcumin on tumor cell growth and invasion, highlighting the regulatory axis. Moreover, CDCA3 was confirmed as a direct target of miR-134-5p, and its knockdown suppressed cancer progression (29). Notably, CDCA3 was found to interact with CDK1, and CDK1 overexpression negated the tumor-suppressive effects of CDCA3 downregulation. *In vivo* experiments supported these findings, as curcumin treatment significantly reduced tumor growth in mice, accompanied by increased miR-134-5p expression and reduced CDCA3 and CDK1 levels. In conclusion, this study provides strong evidence that curcumin exerts its anticancer effects in CRC by modulating the miR-134-5p/CDCA3/CDK1 axis, thereby suppressing tumor cell proliferation and invasion and promoting apoptosis (29).

#### 3.1.6 The Wnt/β-catenin pathway

The Wnt signaling pathways play a significant role in cancer development by modulating various processes such as cancer cell growth, differentiation, programmed cell death, cell cycle advancement, adhesion, and the formation of blood vessels (30). It is thought that the critical factor in Wnt signaling pathway-related cancer formation is the inhibition of beta-catenin degradation. This leads to the accumulation of free β-catenin in the cytoplasm, which then interacts with Tcf/Lef transcription factors in the nucleus, resulting in the activation of oncogenic target genes like cyclin D1 and c-myc. Research has indicated that Wnt1 serves as a downstream target for microRNAs. Consequently, *in vitro* experiments were carried out to determine if CUR affects this signaling or not (30). Using a xenograft mouse model, SW480 colon cancer cells were implanted into mice, and the effects of curcumin treatment on tumor growth and survival were evaluated (31). Curcumin significantly inhibited tumor growth *in vivo*, and analysis revealed that this effect was primarily due to the suppression of cell proliferation, rather than the induction of apoptosis. Mechanistic investigations showed that curcumin suppressed the Wnt/β-catenin signaling pathway, a key regulator of proliferation and oncogenesis in CRC (31). Specifically, curcumin treatment led to reduced expression of β-catenin, Axin, and TCF4 in SW480 cells. Further analysis of microRNA expression revealed that miR-130a was downregulated by curcumin. Functional studies demonstrated that overexpression of miR-130a reversed the antiproliferative effects of curcumin, indicating that miR-130a is a critical mediator in the curcumin-regulated inhibition of the Wnt/β-catenin pathway. These findings suggest that curcumin exerts its antitumor effects in colon cancer at least in part by suppressing miR-130a, thereby downregulating Wnt/β-catenin signaling and inhibiting tumor cell proliferation (31).

#### 3.1.7 Synergy with chemotherapy

Another perspective for CUR's effects is how it can decrease the resistance of cancer cells to chemotherapeutic drugs through regulating epigenetics. In this regard, Fan et al. worked on a cisplatin-resistant cell line (HT29) and found that Results showed that curcumin significantly synergized with cisplatin (combination index <1) to suppress the proliferation of CRC cells more effectively than either agent alone (32). Researchers observed a marked increase in glutamine metabolism, revealing a glutamine-addictive phenotype in resistant CRC cells. Curcumin treatment was found to inhibit glutamine metabolism, and interestingly, under low-glutamine conditions, colon cancer cells exhibited reduced sensitivity to curcumin, indicating a functional link between glutamine availability and curcumin efficacy (32). Further mechanistic insights were gained through a miRNA microarray analysis, which identified that miR-137, a known tumor suppressor in CRC, was significantly upregulated by curcumin. Bioinformatic predictions and luciferase reporter assays confirmed that miR-137 directly targets the 3′ untranslated region (UTR) of GLS messenger RNA (GLS mRNA), the gene encoding glutaminase, a key enzyme in glutamine metabolism (32). Functional rescue experiments demonstrated that miR-137-mediated suppression of GLS was critical for curcumin-induced cisplatin sensitization. In summary, curcumin was able to reverse cisplatin resistance in CRC cells by upregulating miR-137, which in turn downregulated GLS and impaired glutamine metabolism. These findings highlight a novel mechanism by which curcumin may enhance chemotherapy response in CRC, acting through the miR-137/GLS axis to target metabolic vulnerabilities in drug-resistant cells (32).

Another similar study also found that curcumin treatment led to the upregulation of tumor-suppressive miR-200 family members, particularly miR-200c, in chemoresistant CRC cells (33). This upregulation was associated with the downregulation of ZEB1, a transcription factor that promotes EMT, thereby reversing EMT and reducing metastatic potential. The combination of curcumin and 5-FU significantly inhibited cell proliferation and induced apoptosis in chemoresistant CRC cells more effectively than either agent alone (33). This synergistic effect suggests that curcumin can sensitize CRC cells to 5-FU chemotherapy. In a mouse xenograft model, the combined treatment of curcumin and 5-FU resulted in a substantial reduction in tumor growth compared to control or single-agent treatments, indicating the potential of this combination therapy in overcoming chemoresistance in CRC (33). Oxaliplatin (L-OHP) resistance represents a significant hurdle in the effective treatment of CRC (34). One of the proposed mechanisms underlying this resistance involves the regulation of excision repair cross-complementing group 1 (ERCC1) by tumor-expressed microRNAs (miRNAs), yet few studies have comprehensively explored which miRNAs contribute to this drug resistance (34). Curcumin, a bioactive compound with known anticancer properties, has been reported to reverse chemotherapy resistance in various cancers. However, its influence on ERCC1 expression and associated miRNA regulation in CRC had not been previously characterized. To investigate this, researchers established an L-OHP-resistant HCT116 CRC cell line (HCT116/L-OHP) (34). Cell viability assays (MTT) confirmed that curcumin, when combined with L-OHP, significantly reduced resistance compared to L-OHP alone. Apoptosis analysis using flow cytometry revealed that the combination treatment also enhanced cell death in resistant cells. At the molecular level, RT-PCR and Western blot analysis showed that curcumin decreased the expression of several key genes associated with drug resistance, including ERCC1, Bcl-2, GST-π, MRP, P-gp, and survivin. Importantly, curcumin's ability to suppress ERCC1 expression was found to be mediated by miR-409-3p, a microRNA upregulated upon treatment. These findings suggest that curcumin can sensitize resistant CRC cells to oxaliplatin by modulating miR-409-3p, which in turn downregulates ERCC1, a gene known to be involved in DNA repair and drug resistance. Thus, curcumin may serve as a promising adjuvant therapy to reverse oxaliplatin resistance in CRC (34).

Despite promising anticancer effects in preclinical studies, curcumin's clinical translation is hindered by poor oral bioavailability, largely due to rapid metabolism, low aqueous solubility, and poor systemic absorption (35). To address these limitations, various strategies have been developed, including the use of curcumin analogs, liposomal encapsulation, polymeric nanoparticles, micelles, and phospholipid complexes such as Meriva^®^, INDENA S.p.A., Milan, Italy (35, 36). These formulations aim to improve curcumin's stability, circulation time, and tissue targeting (36). Several early phase clinical trials have assessed the safety and efficacy of curcumin in CRC patients. For example, a phase I/II study (37) demonstrated that oral curcumin at doses up to 3.6 g/day was well tolerated and associated with reduced oxidative DNA damage in malignant colorectal tissues. More recent trials have evaluated curcumin in combination with standard chemotherapy (e.g., FOLFOX), with some showing improved tolerability and a modest enhancement in response markers; however, large-scale efficacy data are still lacking. These findings support further investigation of optimized curcumin formulations in well-designed, controlled clinical trials (37).

### 3.2 Resveratrol (RSV) affects CRC cells through epigenetic processes

RSV is a natural polyphenol compound produced by plants, such as grapes, blueberries, and peanuts, as a defense against injury or pathogens. Pharmacologically, RSV interacts with various biological targets but suffers from very low oral bioavailability due to rapid metabolism in the liver and intestines, limiting its effectiveness. Chemically, it exists in two isomeric forms (*cis* and trans), with the *trans* form being more stable. Plants synthesize RSV via specific enzymes, and certain microbes can modify it into other compounds with altered activity. In addition to its epigenetic and anticachectic properties, resveratrol exerts direct antitumor effects in CRC through multiple mechanisms. It has been shown to induce apoptosis by activating pro-apoptotic proteins (e.g., Bax, p53) and inhibiting antiapoptotic factors (e.g., Bcl-2) (38). Resveratrol also suppresses the NF-κB pathway, a key regulator of inflammation and cell survival, thereby reducing the transcription of oncogenes and cytokines involved in tumor progression. Furthermore, it inhibits tumor angiogenesis by downregulating vascular endothelial growth factor (VEGF) and matrix metalloproteinases (MMPs), impairing the neovascularization essential for tumor growth and metastasis. These complementary actions enhance the therapeutic potential of resveratrol as an adjunctive agent in CRC management (38–40). In summary, while RSV is well studied and shows some biological activity in lab settings, robust clinical evidence supporting its health benefits or therapeutic use in humans is lacking. Studies on epigenetic processes show that mainly DNA methylation and microRNAs are the targets of RSV, while the number of studies on histone modifications is not very limited (studies are summarized in Table 2).

**Table 2:** A summary of studies investigating the effects of resveratrol on epigenetic processes.

**Epigenetic process**	**Model**	**Cell line/animal**	**Result(s)**	**References**
microRNA regulation	*In vitro*	HCT116 and HT29	Up-regulating *miR-34c* which knocked down its target KITLG, and the effect was enhanced in the presence of p53, probably through inactivating the PI3K/Akt pathway	(47)
	*In vivo*	Mouse xenografts	*miR-34c* level was elevated in xenografts of Res-treated mice, while the KITLG was decreased	(47)
	*In vitro*			(48)
	*In vitro*	HCT116	Promoting apoptosis and inhibiting invasion by upregulating miR-200c and modulating EMT	(41)
	*In vitro*	SW480	Resveratrol upregulates miR-663, a tumor-suppressor miRNA that specifically targets *TGFβ1* transcripts	(42)
	*In vitro*	NCM-460 and HCT8, HCT116, RKO, SW480, and SW620	Resveratrol upregulated miR-125b-5p by increasing its stability and suppressed TRAF6-induced signal pathway in a dose/time-dependent manner	(44)
	*In vivo*	Female BALB/c non-thymus (NU/NU) nude mice aged 3–5 weeks	Increasing miR-125b-5p and reducing metastasis	(44)
	*In vivo*	Mice	Resveratrol treatment increased the expression of miR-96, a microRNA known to inhibit *Kras* translation	(43)
	*In vitro*	NCM460, LoVo, and SW480	Upregulating miR-769-5p and downregulating MSI1	(46)
DNA methylation	*In vitro*	HCT116, CO115, and SW48	Resveratrol induced the expression of p53, cleaved caspase-3, and PARP	(50)
	*In vitro*	HT116 and SW480	Modulating ZEB1 expression and m6A RNA methylation, and decreasing EMT and invasion	(51)
Histone modification	*In vitro*	-	RSV stabilizes the CARM1 protein, leading to its upregulation and enhanced methyltransferase activity	(49)

#### 3.2.1 microRNAs

There are numerous studies on the roles of RSV in regulating the expression of a diverse range of microRNAs. A key molecular factor in CRC progression is miR-200c, which is known to suppress tumor development by inhibiting EMT. A recent study investigated the potential of RSV, a polyphenolic compound found in red wine, to influence the behavior of CRC cell (41). Results of this study demonstrated that RSV significantly reduced the viability of HCT116 cells. Silencing miR-200c led to increased expression of mesenchymal markers, such as vimentin and ZEB1, alongside decreased E-cadherin expression, enhanced cell migration, and reduced apoptosis—all indicators of EMT induction. Remarkably, treatment with RSV counteracted these effects, restoring miR-200c expression and reversing the EMT phenotype, suggesting a shift toward mesenchymal-to-epithelial transition (MET) (41). In conclusion, this study highlights the critical role of RSV in promoting apoptosis and inhibiting invasiveness in CRC cells by upregulating miR-200c and modulating EMT-related pathways. These findings support the therapeutic potential of RSV in managing CRC progression (41). These promising findings are based on *in vitro* assays in HCT116 cells; however, further *in vivo* validation and clinical studies are required to confirm RSV's potential in reversing EMT through miR-200c modulation in CRC patients.

The transforming growth factor-beta (TGF-β) signaling pathway plays a complex and context-dependent role in CRC. In early stages of tumor development, TGF-β functions as a tumor suppressor by inhibiting cell proliferation, inducing apoptosis, and maintaining epithelial homeostasis. However, as CRC progresses, cancer cells often acquire resistance to these inhibitory effects and instead exploit TGF-β signaling to promote tumor growth and malignancy. In advanced CRC, the TGF-β pathway facilitates EMT. This pro-tumorigenic shift is mediated through SMAD-dependent and SMAD-independent signaling cascades, which regulate the expression of genes involved in cell migration, immune evasion, and extracellular matrix remodeling. As such, the dual nature of the TGF-β pathway in CRC, suppressive in early stages and promotive in later stages, makes it a critical focus of investigation for targeted therapeutic interventions. In this regard, several studies have focused on the role of RSV in modulating the TGF-β signaling pathway through miRNAs. Findings reveal that RSV significantly downregulates several oncogenic miRNAs that normally inhibit the expression of tumor suppressor genes such as Dicer1, PDCD4, and PTEN, as well as critical elements of the TGF-β signaling pathway (42). Concurrently, RSV upregulates miR-663, a tumor-suppressor miRNA that specifically targets TGFβ1 transcripts. Interestingly, RSV also enhances the expression of TGF-β receptor types I and II (TGF-βR1 and TGF-βR2), yet paradoxically reduces the transcriptional activity of SMAD proteins, the principal mediators of canonical TGF-β signaling (42). This dual action suggests a nuanced regulatory effect, whereby RSV supports upstream signaling while dampening downstream pro-oncogenic transcriptional responses. Overall, these results underscore the possibility that RSV's anticancer efficacy may, in part, stem from its ability to remodel cellular miRNA profiles. The targeted modulation of miRNAs like miR-663 presents a promising strategy to enhance the compound's antimetastatic and tumor-suppressive properties (42). Although these results shed light on RSV's nuanced regulatory effects on the TGF-β pathway via miRNA modulation, they are primarily derived from cell-based models. Further research is warranted to establish these interactions *in vivo* and evaluate their clinical significance.

A critical step in the progression of CRC involves activating mutations in the Kras gene, which drives tumor development. Therefore, strategies that prevent the proliferation of cells harboring these mutations are vital for effective CRC prevention (43). To investigate RSV's efficacy in CRC prevention and treatment, researchers employed a genetically engineered mouse model in which APC was knocked out and Kras was specifically activated in the distal colon, simulating sporadic CRC (43). When mice were fed an RSV-enriched diet at concentrations equivalent to 105 or 210 mg per day in human doses, prior to the visible development of tumors, a significant 60% reduction in tumor incidence was observed. Interestingly, in the minority of mice (40%) that developed tumors, Kras expression was absent, suggesting that RSV-mediated suppression of Kras-driven tumor growth (43). In a therapeutic setting where mice were first allowed to develop tumors before initiating RSV treatment, remarkable outcomes were observed. One-third of the mice exhibited complete tumor remission, while the remaining mice showed a 97% reduction in average tumor size. Molecular analysis revealed that RSV treatment increased the expression of miR-96, a microRNA known to inhibit Kras translation, highlighting a potential epigenetic mechanism underlying its antitumor effects (43). These findings suggest that RSV may act as both a preventative and therapeutic agent in CRC by downregulating Kras expression through the upregulation of miR-96, ultimately disrupting key pathways involved in tumor growth and progression (43). While this study provides compelling *in vivo* evidence of RSV's chemopreventive potential in a Kras-driven mouse model, further clinical investigations are needed to assess its efficacy and dosing strategies in human populations.

Recent research highlights RSV as a promising agent in suppressing CRC metastasis, although the precise molecular mechanisms remain incompletely understood. One study identifies miR-125b-5p as a tumor-suppressive microRNA that is significantly downregulated in CRC tissues and cell lines (44). Its expression was found to inversely correlate with TNF receptor-associated factor 6 (TRAF6), a direct target of miR-125b-5p. Functional analyses revealed that overexpression of miR-125b-5p or silencing of TRAF6 markedly inhibited the metastatic behavior of CRC cells (44). Importantly, RSV treatment elevated miR-125b-5p expression by enhancing its stability and concurrently downregulated TRAF6-associated signaling. These molecular effects translated into substantial reductions in CRC cell migration and invasion (44). *In vivo* models further validated these findings: suppression of miR-125b-5p promoted CRC lung metastases, whereas RSV treatment effectively hindered this process. Overall, the study delineates a novel antimetastatic mechanism in which RSV inhibits CRC progression through the activation of the miR-125b-5p/TRAF6 axis, highlighting its therapeutic potential, especially in patients with diminished miR-125b-5p expression (44). Despite *in vivo* validation in murine models, translating these findings to clinical settings remains a challenge. Well-designed human trials are necessary to determine the therapeutic relevance of RSV-mediated miR-125b-5p upregulation.

Some studies that have examined the anti-inflammatory effects of RSV. For instance, Altamemi et al. used the Apc^*Min*^/+ mouse model and induced inflammation-driven tumor formation in them (45). Simultaneously, mice were administered RSV over 5 weeks. RSV-treated mice exhibited significantly fewer and smaller polyps compared to the control group, alongside reduced histological evidence of epithelial damage and lower proliferation of intestinal epithelial cells (45). Immunological analysis revealed that RSV treatment attenuated the DSS-induced increases in inflammatory immune cell populations, including cluster of differentiation 4 (CD4+) and CD8+ T cells, B cells, natural killer T (NKT) cells, and myeloid-derived suppressor cells in mesenteric lymph nodes. Furthermore, RSV significantly reduced levels of pro-inflammatory cytokines, particularly interleukin-6 (IL-6) and tumor necrosis factor-alpha (TNF-α), as well as decreased expression of cyclooxygenase-2 (COX-2) (45). Microarray profiling identified 104 miRNAs whose expression was altered by RSV treatment, with two miRNAs, miR-101b and miR-455, as being significantly upregulated. Both are known to possess anti-inflammatory functions. Pathway analysis indicated that these differentially expressed miRNAs likely target mRNAs involved in inflammation and colorectal tumorigenesis (45).

Another study sought to elucidate the role of RSV in regulating tumor-related behaviors in CRC, with a focus on its interaction with the miR-769-5p/MSI1 signaling pathway (46). Using both *in vivo* and *in vitro* models, researchers investigated the impact of RSV on cell viability, proliferation, apoptosis, and migration (46). QRT-PCR revealed that miR-769-5p expression was significantly reduced, while the expression of Musashi RNA-binding protein 1 (MSI1), a known oncogene, was elevated in CRC tissues and cells compared to controls. Treatment with RSV reversed these trends, upregulating miR-769-5p and downregulating MSI1. RSV inhibited the proliferation and migration of CRC cells while promoting apoptosis. The dual-luciferase reporter assay confirmed that MSI1 is a direct target of miR-769-5p. Importantly, overexpression of MSI1 weakened the antitumor effects of miR-769-5p, affirming its role as a downstream effector (46). *In vivo*, overexpression of miR-769-5p enhanced the tumor-suppressive effects of RSV in mice, leading to reduced tumor growth.

A study tried the combination of RSV and Oxa on HCT116 and HT29 cell lines, focusing on the tumor suppressor microRNA miR-34c (47). They used 100 μM RSV for HT29 cells and 50 μM RSV for HCT116 cells and found that RSV inhibited the viability, proliferation, migration, and invasion of CRC cells, while promoting apoptosis. RSV decreased proliferation by 45.9% in HT29 cells and 57.4% in HCT116 cells (47). Also, RSV increased apoptosis in HT29 cells by 38.6% and in HCT116 cells by 45.5%. In addition, RSV significantly suppressed cell migration by 42.2% and invasion by 51.1% in HCT116 cells. These effects were partly mediated by upregulating miR-34c, which suppressed its target gene KITLG, with more potent effects observed in p53-positive cells, likely due to the inhibition of the PI3K/Akt pathway. RSV also sensitized CRC cells to oxaliplatin in a miR-34c-dependent manner (47). The mechanism behind the upregulation of miR-34c involved decreased methylation of its promoter. In mouse xenograft models, both RSV and Oxa reduced tumor growth, and their combined treatment had enhanced efficacy. Tumors from RSV-treated mice showed increased miR-34c and decreased KITLG expression, accompanied by reduced IL-6 levels, suggesting that RSV may disrupt IL-6-driven tumor progression. In summary, RSV suppresses CRC by activating the miR-34c/KITLG pathway, especially in the presence of p53, and enhances oxaliplatin sensitivity through this mechanism. Additionally, Res-induced miR-34c may inhibit IL-6-mediated cancer progression (47).

There are also some studies that combine RSV with other natural products for achieving a better conclusion. For instance, a study explores the synergistic anticancer effects of RSV and quercetin (RQ) on HT29 colon cancer cells (48). Administered in a 1:1 ratio, RQ significantly reduced cell viability, indicating potent cytotoxic effects. The combination also decreased ROS production by up to 2.25-fold and enhanced antioxidant capacity by up to 3fold, suggesting a role in mitigating oxidative stress (48). Mechanistically, RQ treatment induced apoptosis by a 2fold increase in caspase-3 cleavage and elevated PARP cleavage (48). Importantly, RQ suppressed the expression of oncogenic microRNA-27a (miR-27a) and upregulated ZBTB10, a zinc finger protein that represses Sp transcription factors (48). Taken together, while the findings offer valuable mechanistic insight and suggest a promising therapeutic strategy, further research, particularly *in vivo* studies and broader mechanistic exploration, is needed to fully validate and expand on these results.

#### 3.2.3 Histone modifications

Cisplatin is effective, but its use is limited due to its side effects and the resistance developed by cancer cells. Enhancing the efficacy of cisplatin while reducing its toxicity is a critical research focus. RSV, at high doses, can be toxic, so a study focuses on non-cytotoxic (low) concentrations of RSV to explore safe combination therapies. In this *in vitro* study, cancer cell lines were treated with cisplatin alone, RSV alone, and combinations of both (49). The combination of RSV at non-toxic levels and cisplatin significantly inhibited cancer cell proliferation more than either agent alone. It was identified that coactivator-associated arginine methyltransferase 1 (CARM1) as a new binding protein for RSV (49). Their findings demonstrated that increasing CARM1 levels enhanced the sensitivity of cancer cells to RSV (49). Notably, they discovered that CARM1 played a crucial role in the sensitivity of cells to cisplatin when induced by RSV. Further molecular investigations indicated that RSV stabilizes the CARM1 protein, leading to its upregulation and enhanced methyltransferase activity. They also observed a significant increase in the methylation levels of H3R17 in the promoter region of p21, a downstream target of CARM1 that is involved in cell cycle arrest, following RSVL treatment. Lastly, the bioinformatics analysis suggested that CARM1 may serve as a promising biomarker for assessing sensitivity to chemotherapeutic agents and for predicting outcomes in cancer patients (49).

#### 3.2.4 DNA methylation

Studies in this field are limited, and the study of Liu et al. (50) in 2019 is one example. These researchers demonstrated that RSV significantly inhibits the viability of various CRC cell lines, including HCT116, CO115, and SW48, suggesting its potential to curb cancer cell proliferation. At the molecular level, RSV was found to upregulate p53 expression and activate its downstream targets, such as BAX and PUMA, which are critical in mediating p53-dependent apoptosis (50). A key finding of the study is that RSV also enhances the expression of SET7/9, a lysine methyltransferase that modifies p53 by monomethylating it at lysine 372 (50). This methylation stabilizes and activates p53, thereby increasing its tumor suppressor functions. The increased expression of SET7/9 following RSV treatment was accompanied by elevated levels of apoptotic markers, including cleaved caspase-3 and poly [adenosine diphosphate (ADP)-ribose] polymerase (PARP), further confirming the induction of apoptosis. To validate the role of SET7/9 in this process, the authors performed knockdown experiments using short hairpin RNA (shRNA) targeting SET7/9 (50). This intervention significantly reduced the RSV-induced expression of p53, cleaved caspase-3, and PARP, indicating that SET7/9 is essential for mediating the apoptotic effects of RSV via p53 activation. A major limitation is the absence of *in vivo* studies, which are crucial to assess whether the observed mechanisms also function in a complex biological environment, such as within a living organism. Another study explores the protective role of RSV against cadmium (Cd)-induced epithelial–EMT in CRC cells, a key process involved in tumor progression and metastasis (51). Cadmium is a toxic environmental pollutant known to promote tumorigenesis through mechanisms including the induction of EMT. EMT is characterized by enhanced cancer cell migration and invasion, typically marked by a loss of epithelial markers, like E-cadherin and a gain of mesenchymal markers such as N-cadherin, vimentin, and transcription factors like ZEB1.

These researchers conducted a series of *in vitro* experiments to examine the interplay between Cd exposure, EMT progression, and the modulatory effects of RSV (51). Using Transwell assays, they demonstrated that Cd significantly enhanced the migration and invasion capabilities of CRC cells in a concentration-dependent manner. Molecular analyses via Western blotting and reverse transcription quantitative polymerase chain reaction (RT-qPCR) revealed that Cd exposure led to upregulation of mesenchymal markers (ZEB1, N-cadherin, vimentin) and downregulation of the epithelial marker E-cadherin, confirming the induction of EMT (51). Subsequent treatment of Cd-exposed CRC cells with varying concentrations of RSV resulted in a reversal of the Cd-induced migratory and invasive phenotype. RSV restored E-cadherin expression while significantly suppressing mesenchymal markers, indicating that it can inhibit EMT progression. These findings suggest that RSV mitigates the pro-metastatic effects of cadmium on CRC cells. To further elucidate the mechanism, the study examined N6-methyladenosine (m6A) RNA methylation, an epigenetic modification that influences mRNA stability and translation. Using methylated RNA immunoprecipitation (Me-RIP) combined with RT-qPCR, the researchers found that RSV treatment altered the m6A modification patterns of EMT-related RNAs, particularly ZEB1 mRNA. This indicates that RSV may exert its EMT-inhibitory effects not only through transcriptional regulation but also by modifying the epigenetic landscape of CRC cells via m6A-dependent mechanisms (51). In conclusion, this study provides strong evidence that cadmium promotes EMT and increases the invasive potential of CRC cells, and that RSV can effectively reverse these malignant changes. It does so by modulating ZEB1 expression and m6A RNA methylation, offering valuable insights into the therapeutic potential of RSV in counteracting environmentally induced cancer progression. While the findings are promising, they are based solely on *in vitro* data. Further *in vivo* studies are needed to validate these mechanisms and confirm the translational relevance of RSV as a chemopreventive or therapeutic agent in CRC. In summary, resveratrol exerts its anticancer effects in CRC through a multifaceted network of molecular mechanisms. These include the modulation of tumor-suppressive and oncogenic microRNAs (e.g., miR-34a, miR-200c, miR-125b-5p), the reversal of aberrant DNA methylation, and the alteration of histone modification patterns, all of which contribute to the epigenetic reprogramming of cancer cells. Additionally, RSV consistently inhibits epithelial–mesenchymal transition (EMT), a key process in tumor invasion and metastasis, by targeting regulatory axes such as miR-200c/EPM5 and miR-34c/KITLG. These concerted actions influence critical signaling pathways, including Wnt/β-catenin, PI3K/Akt, and TGF-β, ultimately suppressing tumor proliferation, invasion, and chemoresistance. Together, these findings underscore RSV's potential as a promising adjunctive or chemopreventive agent in CRC therapy, particularly through its capacity to epigenetically modulate cancer-related gene expression and metastatic behavior.

Although resveratrol is generally regarded as safe, especially at dietary levels, high pharmacological doses may exert off-target or pro-oxidant effects, underscoring the need for careful dose optimization in future clinical applications. Despite its promising anticancer mechanisms, resveratrol suffers from poor oral bioavailability due to rapid metabolism, low aqueous solubility, and limited systemic absorption (52). These pharmacokinetic limitations pose significant challenges for clinical translation. To address this, various delivery platforms, including liposomes, polymeric nanoparticles, solid lipid nanoparticles, and phospholipid complexes, have been developed to enhance resveratrol's stability, absorption, and tissue targeting (52). These advanced formulations have shown improved bioavailability and therapeutic efficacy in preclinical models, offering a promising avenue to optimize resveratrol-based interventions for CRC (52).

## 4 Epigenetic and chemopreventive actions of EGCG and green tea derivatives in CRC

**Figure 2:**
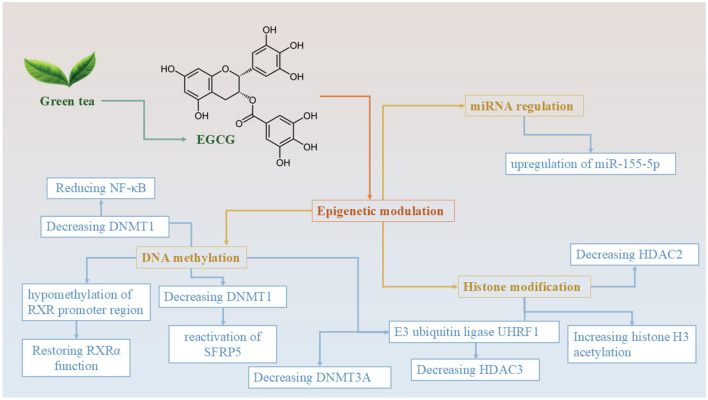
Epigenetic mechanisms of EGCG derived from green tea in colorectal cancer prevention and therapy. This diagram illustrates the multifaceted epigenetic regulatory roles of epigallocatechin-3-gallate (EGCG), the major catechin found in green tea. EGCG modulates gene expression and cancer-related pathways through three major epigenetic mechanisms: DNA methylation, histone modification, and miRNA regulation. In terms of DNA methylation, EGCG reduces the levels of DNMT1 and DNMT3A, leading to hypomethylation of the *RXR*α promoter region and reactivation of tumor suppressor genes, such as *SFRP5*. This cascade results in the downregulation of NF-κB signaling and the restoration of normal retinoid signaling. Through histone modification, EGCG inhibits the E3 ubiquitin ligase UHRF1, thereby reducing the activity of HDAC3. Additionally, EGCG downregulates HDAC2, which enhances histone H3 acetylation–a marker associated with open chromatin structure and active gene transcription. EGCG also influences miRNA expression, specifically by upregulating tumor-suppressive microRNA *miR-155-5p*, which plays a role in modulating oncogenic pathways. Collectively, these epigenetic effects contribute to EGCG's anticancer potential, making it a promising agent for colorectal cancer chemoprevention and adjunctive therapy.

Green tea, derived from the leaves of *Camellia sinensis*, is one of the most widely consumed beverages globally and has been recognized for its health-promoting properties (53). Its relevance to CRC stems from its rich composition of bioactive compounds, including flavonoids, alkaloids, and amino acids that contribute to its anti-inflammatory, antioxidant, and antitumor effects. Green tea is well tolerated, has low toxicity, and is readily incorporated into the daily diet, making it a practical candidate for long-term chemoprevention. Emerging evidence suggests that green tea and its components can modulate epigenetic processes, immune responses, and key signaling pathways involved in CRC progression, supporting its potential role in integrative cancer prevention strategies (53). Green tea is rich in polyphenols, particularly catechins, which are flavanols known for their antioxidant properties. The most abundant catechins are EGCG, epigallocatechin (EGC), epicatechin-3-gallate (ECG), and epicatechin (EC). Green tea contains much higher concentrations of these polyphenols compared to black or oolong tea. EGCG is an ester formed from epigallocatechin and gallic acid, classified as a type of catechin. Certain fruits (like apples and berries) and vegetables (such as onions and kale) contain catechins, though in lower amounts compared to tea. As the most prevalent catechin found in tea, EGCG is a polyphenol currently being studied for its possible impacts on human health and various diseases (Figure 2). It is commonly included in numerous dietary supplements. From a DNA methylation point of view, reduction in promoter methylation utilizing EGCG restores retinoid X receptor alpha (RXRα) expression in human colon cancer (54). RXRα is a nuclear receptor that plays a crucial role in regulating gene expression and maintaining normal cellular functions. In CRC, RXRα expression is frequently downregulated due to hypermethylation of its promoter region—an epigenetic modification that inhibits transcription and contributes to tumor progression. Reversing this epigenetic silencing and restoring RXRα function could have therapeutic implications (54). To investigate this, the researchers treated human colon cancer cell lines with EGCG and analyzed both RXRα expression and the methylation status of its promoter. They used quantitative real-time PCR to measure mRNA levels of RXRα and bisulfite sequencing to evaluate promoter methylation patterns. The results showed that EGCG treatment significantly increased RXRα mRNA expression, which correlated with a marked reduction in methylation levels at the RXRα promoter. This suggests that EGCG can induce promoter demethylation, thereby reactivating the transcription of RXRα in colon cancer cells. In conclusion, the study provides evidence that EGCG can reverse epigenetic silencing of RXRα by reducing promoter methylation. These findings highlight a potential therapeutic role for EGCG in CRC by targeting epigenetic regulation mechanisms (54).

In addition, researchers have demonstrated that ECGC also affects the expression of DNA methyltransferases (DNMTs). A study using an azoxymethane (AOM)-induced rat model of CRC investigated the chemopreventive potential of these agents, both independently and in combination (55). The experimental setup included 4-week-old male Sprague–Dawley Rats assigned diets containing either green tea extract (0.5%), selenium in the form of selenium-enriched milk protein (1 ppm), or both (55). The results demonstrated that the combination of selenium and green tea had a significantly greater inhibitory effect on the formation of large aberrant crypt foci (ACFs) and tumor parameters than either compound alone (*p* < 0.01). Notably, the combination diet decreased expression of DNA methyltransferase 1 (DNMT1) and increased histone H3 acetylation. These changes were associated with the reactivation of tumor-suppressor gene SFRP5 in normal-appearing colonic tissue (55). Furthermore, the combined treatment reduced β-catenin nuclear localization, cyclin D1 expression, and cellular proliferation (55). Another similar study also worked on the roles of EGCG on DNMTs. The findings revealed that EGCG significantly reduced the protein expression of DNMTs and histone deacetylases (HDACs), specifically DNMT3A and HDAC3, in methylation-sensitive HCT 116 colon cancer cells (56). In contrast, the methylation-insensitive HT29 cell line displayed more stable levels of these proteins after treatment, indicating a selective effect based on epigenetic sensitivity. The reduction in DNMT3A and HDAC3 levels in HCT 116 cells was partially attributed to EGCG's interference with the E3 ubiquitin ligase UHRF1, which typically stabilizes these proteins (56). These results suggest that EGCG disrupts the epigenetic regulatory machinery more effectively in methylation-sensitive colon cancer cells. Therefore, combining EGCG with other DNMT and HDAC inhibitors may offer a targeted therapeutic strategy, particularly for patients whose tumors exhibit heightened sensitivity to DNA methylation-based silencing (56). One emerging epigenetic marker in this context is the methylation status of repetitive long interspersed nuclear elements-1 (LINE-1), which plays a pivotal role in both carcinogenesis and chemoprevention. In a recent *in vivo* study, green tea extract (*C. sinensis*) was shown to largely prevent carcinogen-induced LINE-1 hypomethylation in liver, spleen, and kidney tissues, suggesting a robust protective effect. For comparative context, other dietary polyphenols, including RSV-enhanced flavonoid extract and Chinese bayberry, were also evaluated; however, green tea demonstrated the most consistent and broad-spectrum methylation-preserving effects across tissues. These results underscore the strong potential of green tea in maintaining epigenetic stability and reducing CRC risk (57). In a recent *in vivo* study, researchers investigated the impact of various dietary extracts, including green tea (*C. sinensis*), Chinese bayberry (*Morella rubra*), a RSV-enhanced flavonoid extract, and coffee (*Coffea arabica*), on LINE-1 DNA methylation patterns in the liver, spleen, and kidney tissues of mice exposed to the carcinogen 7,12-dimethylbenz[a]anthracene (DMBA) (57). The findings revealed that DMBA-induced hypomethylation was largely prevented by all tested substances, except for Chinese bayberry extract in the kidneys. Notably, the flavonoid extract not only prevented hypomethylation but also induced significant hypermethylation in liver tissues, suggesting a potent chemopreventive effect. These extracts, known for their antioxidant and anti-inflammatory properties, were found to enhance glutathione levels and antioxidant enzyme activity, thereby mitigating free radical damage induced by 7,12-dimethylbenz[a]anthracene (DMBA) (57). This protective mechanism likely involves the activation of DNA methyltransferases and the inhibition of procarcinogen-activating enzymes such as CYP1A1 in the liver. Interestingly, myricetin, a major component of the bayberry extract, exhibited a paradoxical pro-oxidant effect in kidney tissue, which may explain its failure to prevent LINE-1 hypomethylation in that organ. These results underscore the potential of LINE-1 DNA methylation status as a biomarker for evaluating the carcinogenic and chemopreventive impact of dietary bioactive compounds (57).

Another study investigates the epigenetic effects of a fruit seed and peel extract rich in polyphenols and flavonoids on DNMTs and histone deacetylases (HDACs) in a mouse model exposed to the carcinogen 7,12-dimethylbenz[a]anthracene (DMBA) (58). High-performance liquid chromatography (HPLC) analysis revealed that the extract contained 1.74 mg/L of trans-resveratrol (trans-RSV) and 2.37 mg/L of trans-piceid. Based on these concentrations, the estimated daily intake for each mouse was ~5.8 μg of trans-RSV and 7.9 μg of trans-piceid. Administration of DMBA significantly increased the expression of HDAC2 in the liver (58). However, treatment with the fruit extract mitigated this effect, restoring HDAC2 expression levels to those comparable with the control group. Similar trends were observed for HDAC3 and HDAC8 in both liver and kidney tissues, indicating the extract's potential in modulating epigenetic markers associated with carcinogenesis. The protective effects of the extract are attributed to its antioxidant and anti-inflammatory properties (58). The polyphenols and flavonoids present in the extract enhance glutathione levels and induce antioxidant enzymes, reducing oxidative stress caused by DMBA. Additionally, these compounds may increase the activity of DNA methyltransferase enzymes, contributing to the maintenance of normal DNA methylation patterns. However, *in vivo* validation is needed to rely on these results (58). There is only one human study in this field that investigates the effects of selenium (Se) supplementation via Brazil nuts and green tea extract (GTE) on biomarkers associated with CRC risk (59). Thirty-two volunteers over the age of 50 with plasma selenium levels ≤ 1.36 μmol/L were randomized into three groups: Brazil nuts group (*n* = 9): Received ~48 μg/day of selenium through six Brazil nuts. GTE group (*n* = 11): Received four capsules of GTE, totaling 800 mg of EGCG, and the combination group (*n* = 12): Received both Brazil nuts and GTE as described above. Participants consuming Brazil nuts, either alone or in combination with GTE, showed significant increases in plasma selenium levels and rectal SePP mRNA expression (59). This suggests enhanced selenium status and potential improvement in antioxidant defenses. An increase in rectal β-catenin mRNA expression was observed in the Brazil nuts and combination groups, indicating potential modulation of the Wnt signaling pathway, which plays a role in cell proliferation and differentiation (59). Participants in the GTE and combination groups exhibited significant reductions in rectal DNMT1 and nuclear factor kappa B (NF-κB) mRNA levels, suggesting anti-inflammatory effects and potential epigenetic modulation. No significant changes were detected in rectal acetylated histone H3 (Ac-H3) or Ki-67 protein expression across any of the groups, indicating that the interventions did not markedly affect histone acetylation status or cell proliferation rates in rectal tissues (59). The study concludes that supplementation with Brazil nuts and/or green tea extract can modulate specific biomarkers associated with CRC risk, particularly those related to selenium status, Wnt signaling, inflammation, and DNA methylation. However, combining both supplements did not produce additional benefits beyond those observed with each supplement individually. These findings support the potential role of dietary components, like selenium and polyphenols, in CRC prevention strategies (59).

In addition to the effects of EGCG on DNA methylation and histone modification, some miRNAs are also known to be affected by this polyphenol. One study investigates the synergistic effects of EGCG in enhancing the sensitivity of CRC cells to 5-FU (60). Their findings demonstrate that EGCG significantly enhances the cytotoxicity of 5-FU in human colon carcinoma cell lines HCT116 and DLD1. Specifically, co-treatment with 50 μM EGCG reduced the half maximal inhibitory concentration (IC50) of 5-FU from 40 ± 4.2 to 5 ± 0.36 μM in HCT116 cells, and from 150 ± 6.4 to 11 ± 0.96 μM in DLD1 cells. Mechanistically, EGCG co-treatment led to increased apoptosis and DNA damage compared to either agent alone, as evidenced by elevated levels of cleaved caspase-3 and PARP, a decrease in antiapoptotic Bcl-2, and an increase in pro-apoptotic Bad (60). EGCG also downregulated glucose-regulated protein 78 (GRP78), an endoplasmic reticulum chaperone involved in chemoresistance. This downregulation activated the NF-κB signaling pathway (2.55 ± 0.05-fold in HCT116 and 2.27 ± 0.08-fold in DLD1), leading to upregulation of miR-155-5p (2.12 ± 0.02-fold in HCT116 and 2.01 ± 0.01-fold in DLD1) (60). The elevated miR-155-5p expression suppressed the multidrug resistance gene MDR1, thereby decreasing drug efflux and allowing for the intracellular accumulation of 5-FU. This cascade potentiated the antitumor efficacy of 5-FU. In conclusion, EGCG functions as a potent chemosensitizer in CRC by modulating the GRP78/NF-κB/miR-155-5p/MDR1 signaling axis, promoting apoptosis and enhancing 5-FU cytotoxicity. These findings provide a promising foundation for developing EGCG as an adjunct in CRC chemotherapy to overcome resistance and improve therapeutic outcomes (60).

Despite promising mechanistic insights, it is important to note that the majority of studies investigating green tea's anticancer effects have been conducted *in vitro* or animal models. While these findings offer valuable preliminary evidence, they may not fully translate to human physiology. To date, only one human study has assessed the effects of green tea extract on CRC risk, reporting modest changes in biomarkers such as DNMT1 and NF-κB expression. Thus, more rigorous clinical trials are needed to validate the chemopreventive potential of green tea in human CRC. While catechins such as epicatechin gallate (ECG), epigallocatechin (EGC), and epicatechin (EC) are also present in green tea and exhibit antioxidant and anti-inflammatory properties, their epigenetic activity remains less well studied compared to EGCG. Current research into their roles in DNA methylation, histone modification, or microRNA regulation is limited, and their contribution to CRC chemoprevention appears to be of secondary importance at this time.

## 5 Isoflavones in CRC: epigenetic and signaling pathway modulation by genistein

Genistein is a phytoestrogen and isoflavone present in soybeans and other legumes. This natural compound resembles mammalian estrogens in structure and has been associated with several health advantages, such as antioxidant and anti-inflammatory effects, as well as potential cancer-preventive properties. Soy protein isolate (SPI) is a highly purified form of soy protein commonly used in nutritional and biochemical studies. One of the key bioactive compounds found in SPI is genistein, a naturally occurring isoflavone that has been widely studied for its potential health benefits, including anticancer properties (61). Genistein constitutes a significant portion of the isoflavones present in SPI, making SPI an important dietary source of this compound. Clarifying this relationship helps contextualize the biological effects observed in studies utilizing SPI, as many of these effects can be attributed, at least in part, to genistein (61). Soy-based phytoestrogens, such as genistein, have garnered significant interest due to their potential role in CRC prevention and treatment. Epidemiological studies suggest differences in CRC incidence between Eastern and Western populations, which may be partly attributed to dietary variations, including higher soy consumption in Eastern diets, highlighting the importance of exploring genistein's biological effects in this context.

In CRC, genistein has numerous effects, and some of which are possible through epigenetic modulations (Table 3). One of the first studies in this field was conducted in 2013 (62). The results of this study show that Histone H3 acetylation (H3Ac) at the promoters of Sfrp2, Sfrp5, and Wnt5a was significantly downregulated by both SPI and GEN in the post-AOM period. This decrease in H3Ac was associated with reduced RNA polymerase II binding, suggesting a transcriptionally repressive chromatin state. SPI and GEN increased the nuclear abundance of histone deacetylase 3 (HDAC3), supporting the mechanism of transcriptional repression via histone deacetylation (62). Trimethylation of histone H3 at lysine 9 (H3K9me3) and phosphorylation of histone H3 at serine 10 (H3S10P), marks associated with transcriptional activation and cell proliferation, were suppressed by both diets (62).

**Table 3:** Studies exploring the effects of genistein and luteolin on epigenetic processes in CRC.

**Polyphenol**	**Epigenetic process**	**Model of study**	**Results**	**Reference**
Genistein	DNA methylation	*In vivo*	SPI and GEN treatment significantly increased DNA methylation at regulatory regions of Sfrp2, Sfrp5, and Wnt5a	(62)
		*In vitro*	Genistein treatment did not affect the methylation status of the DKK1 promoter	(63)
		*In vitro*	GEN treatment led to a clear decrease in DNA methylation of the Wnt5a promoter	(64)
	Histone modification	*In vivo*	Suppressed Trimethylation of histone H3 at lysine 9 (H3K9me3) and phosphorylation of histone H3 at serine 10 (H3S10P)	(62)
		*In vitro*	Genistein significantly enhanced the acetylation of histone H3 at the DKK1 promoter	(63)
	miRNA	*In vitro*	Genistein downregulated the expression of miR-95 as well as the mRNA levels of Akt and SGK1	(65)

From a DNA methylation point of view, SPI and GEN treatment significantly increased DNA methylation at regulatory regions of Sfrp2, Sfrp5, and Wnt5a, as assessed by methylation-specific PCR and bisulfite sequencing. Bisulfite sequencing revealed that GEN induced methylation of a CpG island in the promoter region of Sfrp5, and this hypermethylated region overlapped with the H3Ac-depleted fragment, indicating cooperative gene repression through both DNA and histone modification (62). The increased DNA methylation and repressive histone marks (decreased H3Ac, increased HDAC3, decreased H3K9me3, and H3S10P) strongly correlated with decreased mRNA expression of the Wnt pathway genes, effectively suppressing aberrant pathway activation caused by AOM. This study identifies a comprehensive epigenetic mechanism by which dietary genistein exerts its chemopreventive effects on the colon during early stage carcinogenesis. The suppression of Wnt pathway activation by GEN involves: Histone deacetylation, Repression of activating histone marks (H3Ac, H3K9me3, H3S10P), Induction of DNA methylation at CpG islands in promoter regions (62). Another study investigates the epigenetic mechanisms through which genistein, with a focus on its regulation of the Wnt signaling pathway antagonist, DKK1. Genistein is known for its ability to block uncontrolled cell growth by modulating signaling pathways, particularly Wnt, and this study aimed to determine whether the genistein-induced upregulation of DKK1 involves changes in DNA methylation or histone modifications (63). To explore this, researchers treated human colon cancer cell lines SW480 and HCT15 with increasing concentrations of genistein. They observed a concentration-dependent reduction in cell proliferation, accompanied by a significant arrest of cells in the G2 phase of the cell cycle. These antiproliferative effects were consistent across several colon cancer cell lines. Further investigation into the role of DKK1 revealed that its overexpression led to pronounced growth suppression, while siRNA-mediated knockdown of DKK1 slightly increased cell proliferation, confirming DKK1's role as a tumor suppressor and a key effector of genistein's action (63).

To understand the mechanism of DKK1 upregulation, the researchers analyzed both DNA methylation and histone modification at the DKK1 promoter region. DNA methylation assays showed that genistein treatment did not affect the methylation status of the DKK1 promoter in any of the tested colon cancer cell lines, suggesting that DNA methylation is not responsible for genistein-induced DKK1 expression. In contrast, chromatin immunoprecipitation assays revealed that genistein significantly enhanced acetylation of histone H3 at the DKK1 promoter in both SW480 and HCT15 cells. This increase in histone acetylation correlated with the elevated expression of DKK1, suggesting that genistein promotes DKK1 transcription by modulating chromatin structure through histone modifications. To further support this mechanism, the study employed trichostatin-A (TSA), a known histone deacetylase inhibitor, which mimicked the effects of genistein on DKK1 expression. This finding confirmed that increased histone acetylation plays a central role in the transcriptional activation of DKK1 by genistein (63). In conclusion, this study demonstrates that genistein inhibits colon cancer cell proliferation and induces G2 phase arrest through upregulation of the Wnt pathway antagonist DKK1. This upregulation is mediated not by changes in DNA methylation, but by enhanced histone H3 acetylation at the DKK1 promoter. These results underscore the potential of genistein as an epigenetic modulator and a promising candidate for the prevention or treatment of CRC through targeted regulation of key tumor suppressor genes (63).

In another similar study, human colon cancer cell lines DLD-1, SW480, and SW1116 were treated with either Novasoy (some soy extract rich in isoflavones) or purified GEN for 4 days. Cell proliferation assays demonstrated that both Novasoy and GEN significantly inhibited the growth of all three cell lines. Following treatment, mRNA expression of Wnt signaling components was assessed using real-time PCR. The data revealed that Wnt5a, a non-canonical Wnt ligand with potential tumor-suppressive functions in colon tissue, was upregulated in a time-dependent manner by both Novasoy and GEN in DLD-1 cells. Interestingly, in SW1116 cells, this induction was specific to GEN, suggesting a cell-line-specific regulatory mechanism (64). To explore whether changes in gene expression were associated with epigenetic modulation, the methylation status of the CpG island in the promoter region of the Wnt5a gene was analyzed using methylation-specific PCR and bisulfite genomic sequencing. Results indicated that GEN treatment led to a clear decrease in DNA methylation of the Wnt5a promoter in SW1116 cells, which correlated with the observed increase in Wnt5a mRNA expression (64). In conclusion, the findings demonstrate that GEN exerts antiproliferative effects in colon cancer cells, at least in part, through the epigenetic regulation of Wnt5a. The induction of Wnt5a expression by GEN is associated with a reduction in promoter methylation, providing mechanistic insight into how dietary isoflavones like GEN can influence gene expression and contribute to the suppression of colorectal carcinogenesis via modulation of the Wnt signaling pathway (64).

In miRNA point of view, research shows that Treatment with genistein led to a significant inhibition of cell proliferation, as measured by MTT assay, and induced apoptosis while on the molecular level genistein downregulated the expression of miR-95, a microRNA implicated in cancer progression, as well as the mRNA levels of Akt and SGK1, two serine/threonine kinases known to promote survival and proliferation in cancer cells. Genistein reduced the expression of total Akt and significantly inhibited its phosphorylation (activation), indicating suppression of the PI3K/Akt signaling pathway, which is frequently upregulated in CRC (65). The antitumor effects of genistein were further validated in an *in vivo* nude mouse xenograft model. Mice treated with genistein showed significantly reduced tumor growth compared to control groups (65). These results collectively suggest that genistein exerts its anti-CRC effects by modulating the Akt signaling pathway and suppressing oncogenic factors such as miR-95 and SGK1. This dual action, inducing apoptosis while inhibiting pro-survival signals, underscores genistein's promise as a natural therapeutic agent in the prevention and treatment of CRC (65). In humans, genistein undergoes rapid phase II metabolism, primarily through glucuronidation and sulfation in the intestines and liver, which significantly reduces its bioavailability. These metabolic transformations limit the systemic concentrations of active genistein, potentially affecting its therapeutic efficacy (66).

## 6 Exercise performance, quality of life, chemotherapy tolerance

Despite recent advancements in CRC treatment, surgery remains a cornerstone of care but is often associated with a significant decline in functional capacity, especially among older patients (67). Postoperative complications contribute to an increase in in-hospital mortality and prolonged hospital stays. Such complications not only have adverse clinical implications but also negatively impact patients' daily quality of life. Lower preoperative health and fitness levels have been identified as predictors of surgical complications and are linked to long-term functional impairments following major surgery in CRC patients. Furthermore, low physical activity levels correlate with poorer prognoses in this population, and functional capacity may deteriorate even before surgery while patients await treatment (68). In light of these concerns, there is an urgent need to develop healthcare strategies that mitigate the adverse effects of surgery on physical function. The presence of multiple risk factors, including the invasiveness of the surgery, baseline physical activity, ASA grade, and age, further elevates the risk of complications. Consequently, preventive interventions targeting these modifiable factors are essential. Prehabilitation, which involves structured interventions before surgery, has emerged as a practical and effective approach to enhance postoperative outcomes (69).

**Figure 3:**
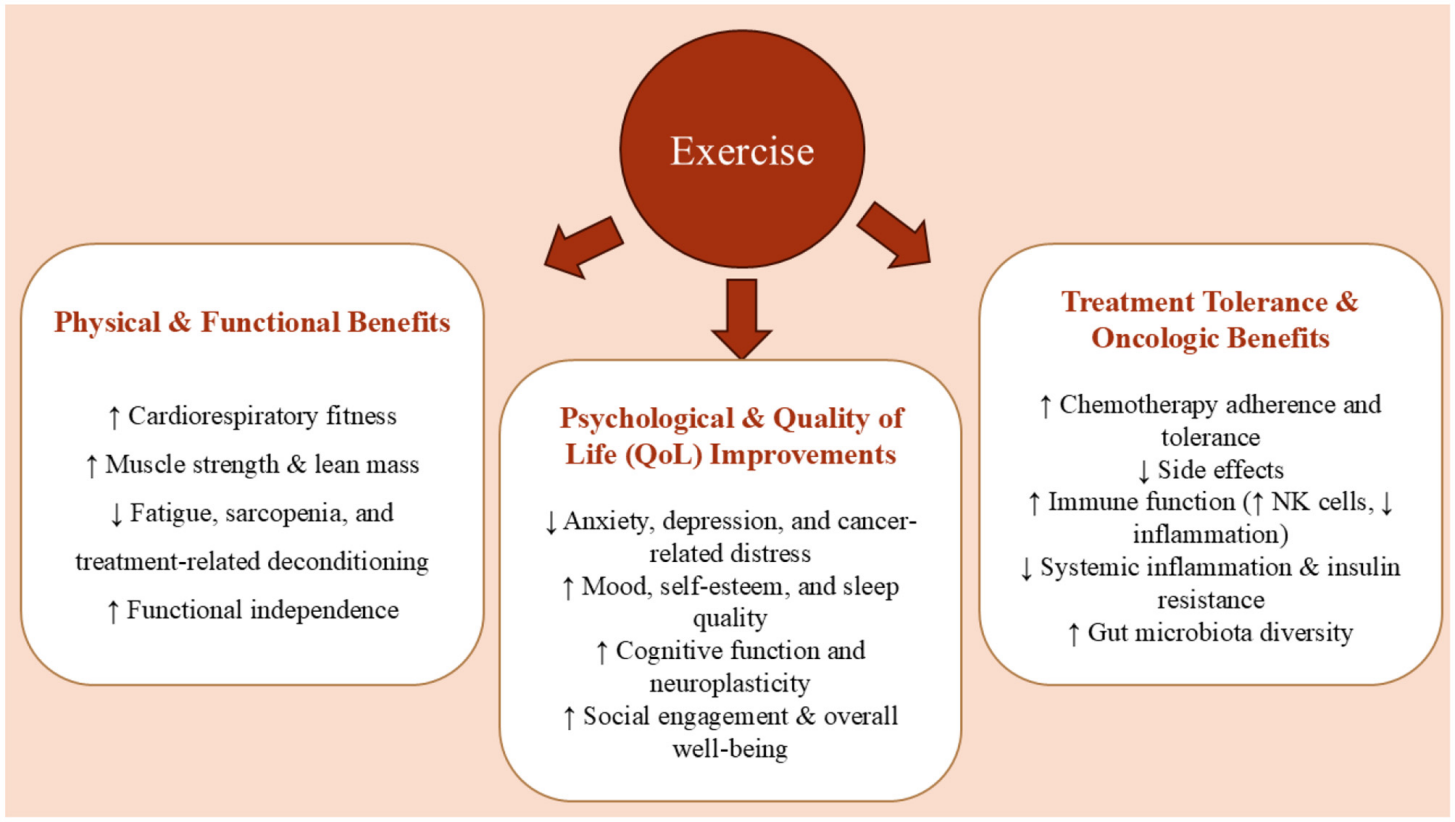
Multifaceted benefits of exercise in colorectal cancer. Physical activity improves exercise capacity, preserves muscle mass, and reduces treatment-related fatigue. It enhances quality of life by alleviating psychological distress and cognitive decline, while also increasing chemotherapy tolerance through improved immune response and metabolic regulation. Together, these effects support better functional recovery and patient resilience throughout the cancer care continuum.

Among these interventions, exercise plays a pivotal role (Figure 3). Exercise is safe for CRC patients. It is associated with a broad spectrum of health benefits, including improvements in quality of life, reduced fatigue and depression, better sleep, enhanced aerobic fitness, increased functional strength, lower body fat, and a reduced risk of mortality (70). Traditionally, aerobic exercises of low to moderate intensity, such as walking, have been recommended as both postoperative rehabilitation and preoperative prehabilitation strategies. The general guideline of 150 min of aerobic activity per week serves as a foundation for these interventions, and resistance exercises performed 2–3 times per week, focusing on major muscle groups such as the legs, chest, back, and core, are recommended to enhance muscular strength (70–73). In the context of CRC care, prehabilitation refers to a set of proactive, multimodal interventions implemented before surgery, to enhance a patient's functional capacity, physiological resilience, and psychological readiness. It typically includes aerobic and resistance exercise, nutritional optimization, and sometimes mental health support (e.g., stress management or counseling). The aim is to improve baseline fitness and minimize the risk of postoperative complications, thereby accelerating recovery. Prehabilitation is especially important in CRC patients, who may have deconditioning or cancer-related fatigue even before treatment begins.

In contrast, rehabilitation is initiated after surgery and focuses on restoring physical function, preventing long-term disability, managing postoperative side effects, and supporting return to daily activities. It may include structured physical therapy, gradual reintroduction of exercise, nutritional counseling, and psychosocial support. While both approaches share similar components, their timing and objectives differ; prehabilitation seeks to prevent or minimize surgical decline, whereas rehabilitation aims to restore or improve function following surgery. An integrated care model that combines both strategies has been shown to improve patient outcomes, including reduced hospital stays, enhanced treatment tolerance, and improved quality of life in CRC survivors. The effectiveness of prehabilitation strategies involving aerobic exercise has been widely evaluated using the six-minute walk test (6MWT), a practical and reliable assessment of functional capacity (74). This measure not only helps determine baseline fitness levels but is also predictive of postoperative outcomes such as disease recurrence and overall survival. Emerging evidence supports the inclusion of both aerobic and resistance training in CRC prehabilitation programs, with concurrent training regimens showing significant improvements in functional capacity within 1–4 weeks. These gains have been documented in numerous studies and are associated with elevated physical activity levels both before and after surgery (74). Importantly, patients who participate in combined aerobic and resistance prehabilitation programs experience better postoperative recovery, a reduction in lean tissue muscle mass loss due to surgical stress, and an overall improvement in their ability to resume daily activities. These findings underline the importance of integrating structured, multimodal exercise interventions into standard CRC care pathways to optimize functional outcomes and enhance long-term survivorship (75, 76).

### 6.1 Combined effects of curcumin and exercise in CRC

Significantly, beyond its widely recognized anticancer effects, curcumin has the potential to help mitigate various side effects associated with chemotherapy. Research indicates that curcumin may reduce symptoms such as nausea, fatigue, and inflammation, which are common challenges faced by cancer patients undergoing treatment. Additionally, curcumin's antioxidant properties can help protect healthy cells from the damaging effects of chemotherapy drugs, potentially improving overall patient wellbeing and enhancing their quality of life during treatment. Its ability to support the immune system may also play a role in helping patients cope with the physical stress of chemotherapy. In a clinical study evaluating the effects of curcumin on chemotherapy side effects, Belcaro et al. investigated multiple cancer types, including colorectal, ovarian, lung, liver, kidney, and stomach cancers. Among 80 patients undergoing chemotherapy, 40 received a daily supplement of 500 mg of curcumin. The results showed that patients in the curcumin group experienced significantly fewer chemotherapy-related side effects, such as nausea, diarrhea, constipation, weight loss, neutropenia, and cardiotoxicity, compared to the control group. Additionally, these patients required fewer medications to manage these adverse effects (77). The 500 mg/day dose of curcumin used in the study falls well within the clinically safe range, as curcumin has been classified as “Generally Recognized As Safe” (GRAS) by the U.S. FDA. Clinical trials have reported good tolerability even at doses up to 8–12 g/day. However, gastrointestinal discomfort can occur at higher levels. In the referenced study, no serious adverse events were reported, and patients receiving curcumin experienced fewer chemotherapy-related side effects compared to the control group, suggesting good tolerability and potential supportive benefits during treatment.

Similarly, turmeric supplementation over 21 days produced a clinically and statistically significant improvement in global health status, symptom scores (including fatigue, nausea, vomiting, pain, appetite loss, and insomnia), and hematological parameters in breast cancer patients treated with paclitaxel (78). Collectively, these findings suggest that incorporating curcumin into standard CRC treatment regimens may help mitigate chemotherapy-induced side effects and enhance patients' quality of life. In addition to its epigenetic and anti-inflammatory properties, curcumin exerts direct antitumor effects in CRC by modulating key oncogenic signaling pathways, such as NF-κB and STAT3, leading to suppressed proliferation, reduced invasion, and enhanced apoptosis. It also enhances chemosensitivity by regulating microRNAs—including miR-21, miR-200c, and miR-34a—which target genes involved in drug resistance and EMT, thereby amplifying the effects of agents, such as 5-fluorouracil and cisplatin. Despite its promising anticancer properties, curcumin's clinical efficacy is significantly limited by its poor oral bioavailability, attributed to its low aqueous solubility, rapid metabolism, and systemic elimination (36). This has prompted extensive research into formulation strategies aimed at enhancing its absorption and therapeutic potential. Approaches include co-administration with bioenhancers, such as piperine (which inhibits hepatic and intestinal glucuronidation), as well as advanced delivery systems, such as liposomes, nanoparticles, micelles, and phospholipid complexes. These technologies have demonstrated improved curcumin plasma concentrations and biological activity in preclinical and early clinical studies, supporting their continued development for CRC therapy (36).

### 6.2 Combined effects of quercetin and exercise in CRC

Although quercetin exhibits anti-inflammatory, antioxidant, and neuroprotective effects that may improve quality of life and reduce cancer-associated symptoms, its direct antitumor efficacy in CRC appears modest. Therefore, its clinical utility may be better positioned as a supportive or adjunctive agent alongside established therapies rather than as a curative treatment on its own.

#### 6.2.1 Depression and neuroprotection

Depression is a common and severe comorbidity among patients with CRC, exacerbating the burden of the disease beyond its physical manifestations. Chronic inflammation and disturbances in brain neurotrophic signaling, particularly involving brain-derived neurotrophic factor (BDNF), are thought to be major contributors to this link between CRC and depression (79). To explore integrative strategies for counteracting CRC-associated depression, a recent study investigated the potential therapeutic effects of quercetin, in combination with exercise training, using a rat model of 1,2-dimethylhydrazine (DMH)-induced CRC (79). The study involved five experimental groups: a control group; a DMH-only group (20 mg/kg subcutaneously, once weekly for 10 weeks); a DMH group followed by quercetin treatment (50 mg/kg orally, once weekly for 12 weeks); a DMH group followed by exercise training for 12 weeks; and a final group receiving both quercetin and exercise training post-DMH induction (79). Behavioral assessments through the open field test and forced swim test showed that DMH-treated rats developed significant depressive-like behaviors, characterized by reduced locomotor activity and increased immobility time, respectively. Histological analysis of the prefrontal cortex revealed neuronal damage and reduced Nissl substance, indicative of neurodegeneration in the DMH group. Furthermore, systemic and localized inflammation was markedly increased, as evidenced by elevated levels of inflammatory cytokines in serum, cortex, and tumor tissues. On a molecular level, DMH exposure significantly downregulated BDNF, its receptor TrKβ, and β-catenin, key proteins involved in neuroplasticity and mood regulation (79). Remarkably, intervention with either quercetin or exercise alone ameliorated several of these pathological alterations. However, the combination of quercetin and exercise training had the most pronounced effects: it reversed depressive-like behaviors, restored neuronal integrity, normalized inflammatory cytokine levels, and significantly upregulated the BDNF/TrKβ/β-catenin signaling pathway in the prefrontal cortex. Importantly, these neuroprotective and antidepressant effects occurred without compromising the antitumor efficacy of DMH (79). In conclusion, this study highlights the synergistic potential of quercetin and physical exercise in mitigating CRC-associated depression. The combined intervention exerts both antidepressive and antitumor effects through the dual action of inflammation suppression and enhancement of neurotrophic signaling, particularly via the BDNF/TrKβ/β-catenin axis. These findings offer promising insights into holistic management strategies for CRC, addressing both physical and psychological dimensions of the disease (79).

#### 6.2.2 Cachexia management

Cancer cachexia, a complex metabolic syndrome characterized by severe body weight, muscle, and fat loss, is a major contributor to cancer-related mortality, particularly in CRC. Despite the lack of approved treatments for this debilitating condition, there is growing interest in nutraceuticals as potential therapeutic agents due to their multiple bioactive properties (80). A study using the Apc(Min/+) mouse model, which closely mimics CRC progression and cachexia seen in humans, was conducted to evaluate the impact of quercetin supplementation on the course of cachexia. Male Apc(Min/+) mice and wild-type C57BL/6 controls, starting at 15 weeks of age, were orally administered 25 mg/kg quercetin or a vehicle solution (Tang juice and water) daily for 3 weeks. The study assessed key clinical and functional outcomes, including body weight, grip strength, neuromuscular performance, and fatigue, as well as biochemical markers of metabolic and inflammatory status (80). Over the treatment period, body weight loss in cachectic Apc(Min/+) mice receiving vehicle (Apc(Min/+)V) was significantly greater (−14% ± 2.3) than in all other groups, including the quercetin-treated Apc(Min/+) mice (Apc(Min/+)Q), which lost significantly less weight (−9% ± 1.3). While not reversing weight loss entirely, quercetin clearly attenuated its severity. Additionally, muscle wasting and loss of grip strength, hallmark features of cachexia, were also significantly mitigated in quercetin-treated Apc(Min/+) mice compared to untreated cachectic controls (80). On the molecular level, cachectic mice treated with vehicle exhibited elevated plasma interleukin-6 (IL-6) and increased phosphorylation of signal transducer and activator of transcription 3 (STAT3) in muscle tissue, both key markers of inflammation and muscle catabolism. These inflammatory indicators were significantly reduced in the quercetin-treated group, indicating that quercetin modulates inflammatory signaling pathways central to cachexia development (80). Notably, the improvement in cachexia-related parameters occurred. These improvements occurred despite no significant reduction in tumor size, indicating that quercetin's benefits were likely due to systemic effects rather than direct tumor suppression. However, quercetin did not improve all aspects of cachexia. No significant benefits were observed in terms of treadmill endurance (runtime to fatigue), hyperglycemia, or hyperlipidemia, suggesting that quercetin's effects may be more localized to skeletal muscle and inflammatory regulation rather than broader metabolic dysfunction (80). In conclusion, this study demonstrates that quercetin supplementation significantly attenuates muscle wasting and inflammatory signaling in a mouse model of CRC cachexia. While it does not fully reverse the condition or correct all metabolic disturbances, quercetin shows promise as a supportive agent in the management of cancer cachexia, justifying further preclinical and clinical research to fully evaluate its therapeutic potential (81). The expression of mucosal proteins plays a crucial role in maintaining the functional integrity of internal organs and the digestive system, particularly in the context of diseases such as colon cancer. Alterations in these protein levels may influence tumor progression, tissue integrity, and organ function, especially in the large intestine where mucosal barriers are vital. This study aimed to investigate the effect of 8 weeks of quercetin supplementation combined with intermittent exercise on the protein levels of intestinal Muc5Ac, Muc4, and polyphosphate in rats with chemically induced colon cancer (81). To conduct the study, 24 rats were randomly divided into four groups: quercetin supplementation, exercise, a combination of quercetin and exercise, and a control group, with six animals in each group. Colon cancer was induced using 1,2-dimethylhydrazine administered over 8 weeks. The quercetin group received daily oral supplementation of 50 mg/kg body weight through gavage. The exercise protocol involved 5 weekly sessions of treadmill running at an intensity of 60–70% of maximum speed (23 m/min), including 2-min rest intervals, for a total duration of 8 weeks. The results demonstrated significant differences in the levels of all three proteins among the groups. Notably, Muc5Ac levels were significantly higher in the group that received both quercetin and exercise compared to the other groups. These findings suggest a synergistic effect of quercetin supplementation and intermittent exercise in enhancing the expression of protective mucosal proteins in the colon (81). In conclusion, this study indicates that the combined intervention of quercetin and intermittent exercise may promote mucosal health by increasing the expression of Muc5Ac and Muc4 in the large intestine of rats with colon cancer, potentially contributing to improved barrier function and resistance to tumor progression (81).

In an effort to explore potential interventions for mitigating the systemic effects of CRC, particularly on the cardiovascular system, one experimental study examined the impact of quercetin supplementation and exercise on oxidative stress markers in heart tissue (82). A total of 80 rats were assigned to either a control group (*n* = 10) or a case group (*n* = 70), in which CRC was chemically induced through weekly subcutaneous injections of 15 mg/kg azoxymethane. The case group was further subdivided into seven experimental groups: untreated patients; saline-treated patients; quercetin-treated patients; and patients undergoing intermittent exercise, continuous exercise, quercetin combined with intermittent exercise, and quercetin combined with continuous exercise. Oxidative stress in the rats' cardiac tissue was assessed by measuring biomarkers, including superoxide dismutase (SOD), catalase (CAT), and malondialdehyde (MDA), using the ELISA method. Data analysis was performed using analysis of variance (ANOVA) via Statistical Package for the Social Sciences (SPSS) software (82). The results indicated a marked increase in oxidative stress in cardiac tissue following the induction of CRC. Notably, quercetin supplementation, both alone and in combination with exercise, significantly elevated the levels of antioxidant enzymes CAT and SOD compared to the untreated and saline groups (*p* < 0.0001). Simultaneously, a significant reduction in MDA levels, a marker of lipid peroxidation and oxidative damage, was observed (*p* < 0.05). Among the interventions, the combination of quercetin with exercise produced the most pronounced improvements in oxidative stress parameters (82). Similar to curcumin, quercetin suffers from poor oral bioavailability, primarily due to its low water solubility, rapid metabolism, and limited intestinal absorption. These pharmacokinetic limitations have hindered its clinical translation despite its promising anticancer properties (83). To overcome this challenge, various formulation strategies have been developed, including the use of nanoparticles, liposomes, micelles, and phospholipid complexes. Co-administration with bioavailability enhancers such as vitamin C or piperine has also shown potential in improving quercetin absorption and systemic exposure. These approaches are being explored to enhance quercetin's therapeutic efficacy *in vivo* and to support its potential use as a chemopreventive or adjunctive agent in CRC (83).

### 6.3 Combined effects of RSV and exercise in CRC

As a potent antioxidant, RSV helps neutralize reactive oxygen species (ROS), thereby protecting cells from oxidative damage, a key contributor to cancer development. Its anti-inflammatory properties stem from its ability to inhibit pro-inflammatory mediators such as NF-κB, IL-6, and TNF-α (84). Additionally, RSV is a known activator of sirtuin 1 (SIRT1), a nicotinamide adenine dinucleotide (NAD^+^)-dependent deacetylase involved in cellular stress resistance, metabolic regulation, and longevity. These bioactivities suggest that RSV may contribute to CRC prevention and therapy by modulating oxidative stress, inflammation, and epigenetic signaling (84). Loss of muscle function due to declining muscle mass and quality is a hallmark of cancer cachexia, a complex syndrome that severely compromises patients' quality of life and survival. The phosphorylation (activation) of mechanistic target of rapamycin complex 1 (mTORC1) and adenosine monophosphate (AMP)-activated protein kinase (AMPK) plays a critical role in regulating energy balance and metabolism, both of which are disrupted in cancer cachexia. Mechanistic target of rapamycin complex 1 (mTORC1) is a key driver of cell growth and protein synthesis; its activation promotes muscle maintenance and anabolic processes. In cachexia, mTORC1 activity is often suppressed, contributing to muscle wasting and weight loss (85). In contrast, AMP-activated protein kinase (AMPK) is a cellular energy sensor that becomes activated under low-energy conditions (86). When AMPK is phosphorylated, it shifts cells toward energy conservation by inhibiting mTORC1 and stimulating fat breakdown and glucose uptake (86). In cancer, AMPK and mTORC1 are often dysregulated, either promoting tumor survival or contributing to systemic energy imbalance. Understanding how compounds like resveratrol influence these pathways helps explain their potential to reduce muscle wasting and impair tumor metabolism (85, 87).

A study investigated the synergistic effects of resistance training and RSV supplementation on cachexia induced by CT26 colon tumors in male mice, focusing on both physiological outcomes and underlying molecular mechanisms (88). Forty-eight mice were randomly assigned to eight experimental groups. Mice in the training groups underwent ladder climbing resistance training for 6 weeks, with weights attached to their tails to simulate progressive overload. RSV-treated groups received a daily oral dose of 50 mg/kg of RSV. In cachectic mice (TSV group), both muscle weight and mTORC1 phosphorylation were significantly reduced compared to healthy controls (HSV), indicating impaired anabolic signaling and muscle atrophy. However, in the TER group (i.e., tumor-bearing mice receiving both exercise and RSV), mTORC1 phosphorylation was markedly increased compared to the TSV, TEV, and TSR groups, demonstrating the synergistic restoration of anabolic signaling (88). Additionally, AMPK phosphorylation, associated with energy stress and catabolic signaling, was highest in HER mice (healthy mice with both exercise and RSV), suggesting enhanced metabolic regulation in healthy conditions. In contrast, in cachectic tumor-bearing mice, the light chain 3 beta II/I (LC3BII/I) ratio, a marker of autophagy activation, was elevated in TSV mice but significantly reduced in TER mice, indicating that the combination therapy reduced muscle degradation processes (88).

In terms of tumor dynamics, all tumor-bearing groups experienced tumor growth. Still, the TER group showed the smallest increase in tumor volume, highlighting the potential antitumor effect of the combined intervention. In tumor tissues, mTORC1 phosphorylation was reduced, and AMPK phosphorylation and LC3BII/I ratios were decreased in the TER group compared to other tumor-bearing groups, suggesting that the combined therapy not only helped preserve muscle mass but also altered tumor metabolic signaling pathways in a favorable direction (88). In conclusion, this study provides compelling evidence that the combination of resistance training and RSV supplementation offers the most potent therapeutic benefit in mitigating cancer cachexia. These effects are strongly supported by molecular findings, such as the upregulation of anabolic pathways (mTORC1) and the downregulation of catabolic and autophagic signaling, as well as reduced tumor progression. This integrative approach represents a promising non-pharmacologic strategy to counteract cachexia and improve clinical outcomes in cancer patients (88). While these preclinical findings offer promising insights into the antitumor effects of resveratrol, particularly in models such as CT26 murine colon cancer, clinical trials in human CRC populations are essential to validate these results and assess therapeutic efficacy and safety. Despite the well-established benefits of exercise for CRC patients, several barriers often hinder consistent participation in exercise. These include advanced age, comorbidities (e.g., cardiovascular or musculoskeletal conditions), treatment-related fatigue, limited motivation, and lack of access to supervised rehabilitation services (89, 90). Such obstacles can reduce engagement with structured exercise programs, particularly during the perioperative or chemotherapy phases of treatment. To overcome these limitations, a shift toward personalized exercise prescriptions, tailored to individual health status, preferences, and treatment stage, is increasingly advocated. Moreover, emerging telehealth and remote coaching platforms offer promising solutions to improve accessibility and adherence, especially for patients in underserved or rural areas. Addressing these barriers is essential for integrating exercise into standard CRC care pathways (89, 90).

## 7 Conclusion

This comprehensive review underscores the multifaceted potential of functional foods, particularly curcumin, RSV, green tea polyphenols (EGCG), quercetin, and genistein, when combined with structured physical exercise, in the prevention and management of CRC. These bioactive compounds exert potent anticancer effects by modulating key molecular and epigenetic pathways, including DNA methylation, histone modification, microRNA regulation, and signaling cascades such as Wnt/β-catenin, PI3K/Akt, NF-κB, and TGF-β. Curcumin, in particular, demonstrates a strong epigenetic footprint, reversing aberrant methylation patterns, restoring tumor suppressor gene expression, and attenuating EMT processes. RSV's efficacy extends through its modulation of miRNAs like miR-200c, miR-125b-5p, and miR-34c, which directly impact metastasis, EMT, and chemoresistance. While curcumin and resveratrol have been the most extensively studied, other bioactive compounds such as quercetin and genistein also show promise, albeit supported by a more limited body of mechanistic and translational evidence. Green tea catechins, especially EGCG, contribute via DNMT and HDAC inhibition, reactivating silenced genes and enhancing chemosensitivity. Genistein and quercetin similarly modify epigenetic markers and inflammatory pathways, and their effects are amplified in synergy with exercise. Exercise further complements these interventions by improving functional capacity, reducing surgical complications, enhancing quality of life, and mitigating CRC-associated depression and cachexia. Prehabilitation programs integrating aerobic and resistance training have been shown to preserve lean muscle mass, improve surgical outcomes, and reduce systemic inflammation. Collectively, the evidence supports a holistic strategy that incorporates functional foods and exercise into clinical care pathways. Such integrative interventions not only target the molecular underpinnings of CRC but also address the psychological and physiological burden of the disease, promoting better survivorship and potentially improving long-term prognosis. Future clinical trials are warranted to validate these findings in human populations and develop precise, personalized lifestyle-based therapies in oncology. Nonetheless, several limitations remain, including the predominantly preclinical nature of existing studies, significant variability in the bioavailability and dosing of phytochemicals such as curcumin and resveratrol, and the lack of standardized clinical protocols for implementing exercise and dietary interventions in oncology settings. While this review highlights the promising effects of various functional foods and natural compounds, including curcumin, resveratrol, EGCG, quercetin, and genistein, in the prevention and therapy of CRC, it is important to emphasize that the vast majority of supporting evidence comes from preclinical studies, including *in vitro* experiments and animal models. These findings offer mechanistic insights into how these agents modulate epigenetic regulation, inflammation, redox balance, and tumor progression; however, direct extrapolation to human patients should be made with caution. Human data remain limited, and only a few clinical studies have investigated these compounds in CRC patients, with mixed or modest results. Therefore, while these bioactive agents hold significant potential as adjunctive or preventive strategies, large-scale, well-controlled clinical trials are needed to confirm their efficacy, safety, and optimal use in CRC management. Although evidence supports exercise as a supportive therapy in cancer care broadly, studies specifically evaluating its role in improving chemotherapy tolerance and adherence in CRC patients are currently lacking, highlighting a critical area for future clinical investigation.
